# Proceedings of the 16th annual conference of INEBRIA

**DOI:** 10.1186/s13722-019-0157-1

**Published:** 2019-09-26

## A1 Active components of a web-based personalised normative feedback: a dismantling study

### Andre Bedendo^1,2^, Jim McCambridge^2^, Jacques Gaume^3^, Altay A. L. Souza^4^, Maria L. O. Souza‐Formigoni^4^, Ana R. Noto^4^

#### ^1^Department of Psychobiology, Universidade Federal de Sao Paulo, Sao Paulo, Brazil; ^2^Department of Health Sciences, University of York, York, United Kingdom; ^3^Alcohol Treatment Centre, Lausanne University Hospital, Lausanne, Switzerland; ^4^Department of Psychobiology, Universidade Federal de Sao Paulo, Sao Paulo, Brazil

##### **Correspondence:** Andre Bedendo - andrebedendo@gmail.com

*Addiction Science & Clinical Practice* 2019, **14(Suppl 1):**A1

**Background:** Web-based Personalised Normative Feedback (PNF) show small to moderate effects on alcohol use among college students. However, little is known about its active components. This study evaluated the effectiveness of two components of PNF in reducing alcohol use and consequences among Brazilian college students.

**Methods:** College students (18–30 years) who reported alcohol use in the last three months (N = 5,476), were included in a three-arm pragmatic randomised controlled trial with 1-, 3-, and 6-month follow-up. Participants were assigned to either: (1) full PNF intervention; (2) Normative feedback (NF) only or (3) Consequences Feedback (CF) only. The primary outcome was AUDIT score; secondary outcomes were number of alcohol-related consequences, drinking frequency, and typical/maximum number of drinks. We used Mixed Models with Multiple Imputation and Pattern-Mixture Model to account for attrition. Post-hoc analysis considered participant interest in knowing more about their drinking.

**Results:** Single component interventions reduced AUDIT score compared to full PNF, with significant effects for NF at 1-month (b = − 0.23, p = 0.048) and for CF at 3-month (b = − 0.33, p = 0.03). Compared to PNF, NF reduced the number of consequences at 1-month (b = − 0.16, p = 0.001) and drinking frequency at 3-month (b = − 0.42, p = 0.03), but increased the number of typical drinks at 6-month (b = 0.38, p = 0.03). CF reduced drinking frequency at 3-month (b = − 0.37, p = 0.045). Attrition models confirmed all results, except for the NF effect on typical drinks and drinking frequency. Post-hoc analyses indicated the superiority of single components effects among those students not interested in knowing more about drinking.

**Conclusions:** Findings suggest that individual components were superior to the full PNF intervention, however this effect was mainly driven by a minority of students (around 20%) who were not interested in receiving it.

**Trial registration:** NCT02058355.

## A2 Optimising the alcohol reduction app, Drink Less

### Claire Garnett^1^, Susan Michie^2^, Robert West^1^, Matt Field^3^, Felix Greaves^4,5^, Matthew Hickman^6^, Eileen Kaner^7^, Marcus Munafo^8^, Robyn Burton^9^, Matthew Walmsley^9^, Jamie Brown^1^

#### ^1^Department of Behavioural Science and Health, University College London, London, UK; ^2^Department of Clinical, Educational and Health Psychology, University College London, London, UK; ^3^Department of Psychology, University of Sheffield, Sheffield, UK; ^4^Public Health England, London, UK; ^5^Department of Primary Care and Public Health, Imperial College London, London, UK; ^6^School of Population Health Sciences, University of Bristol, Bristol, UK; ^7^Institute of Health & Society, Newcastle University, Newcastle, UK; ^8^School of Psychological Science, University of Bristol, Bristol, UK; ^9^Public Health England, London, UK

##### **Correspondence:** Claire Garnett - c.garnett@ucl.ac.uk

*Addiction Science & Clinical Practice* 2019, **14(Suppl 1):**A2

**Background:** Drink Less is an evidence-based smartphone app for reducing excessive drinking in the UK. The development and initial evaluation of Drink Less followed the first two steps of the Multiphase Optimisation Strategy (MOST): (i) identification of intervention components and (ii) randomised factorial screening trial to evaluate the five individual components. The next step in MOST is to develop an optimised version of Drink Less.

**Methods:** The optimisation will be informed by three work packages. First, use of Bayes Factors to analyse additional data collected from extended recruitment of the randomised factorial screening trial. Secondly, an update of the 2017 Cochrane review on digital alcohol interventions and meta-regression of the intervention components associated with effectiveness. Thirdly, a content analysis of Drink Less user feedback received via emails and app store reviews.

**Results:** The Bayes Factors analysis of the factorial trial indicated that one of the five the intervention components (‘Identity Change’) should be removed in the optimised version of the app. The updated meta-regression of the Cochrane review indicated that ‘Behaviour substitution’ and ‘Information about antecedents’ should be introduced into an optimised Drink Less. The content analysis of user feedback identified high priority changes within existing components: customisable drink volumes; ability to update normative feedback; drinking calendar to start on Monday; bug fix relating to time zone changes; clarify how to edit drinks entries and how to navigate to the mood diary.

**Conclusions:** Using a mixed methods approach to optimise Drink Less has provided us with different insights: how to improve the likely effectiveness of the intervention and also providing users with what they want from the intervention, which is crucial for engagement with any intervention. This optimised version will undergo user testing to improve its usability and then the optimised version will be evaluated in a definitive trial.

## A3 Skills training for reducing risky alcohol use in app form among internet help-seekers: a pilot study

### Anne H. Berman^1^, Olof Molander^1^, Miran Tahir^2^, Philip Törnblom^2^, Mikael Gajecki^1^, Kristina Sinadinovic^1^, Claes Andersson^3^

#### ^1^Centre for Psychiatry Research, Department of Clinical Neuroscience, Karolinska Institutet, & Stockholm Health Care Services, Stockholm County Council, Stockholm, Sweden; ^2^Department of Psychology/Centre for Psychiatry Research, Department of Clinical Neuroscience, Karolinska Institutet, & Stockholm Health Care Services, Stockholm County Council, Stockholm, Sweden; ^3^Department of Criminology, Malmö University, Malmö, Sweden

##### **Correspondence:** Anne H. Berman - anne.h.berman@ki.se

*Addiction Science & Clinical Practice* 2019, **14(Suppl 1):**A3

**Background:** Problematic alcohol use in Sweden occurs among 16% of the adult population. Digital interventions of varying intensity have shown positive effects in contributing to reductions in problematic use, and the TeleCoach app has shown positive effects in non-treatment-seeking university students with excessive drinking. This pilot study evaluates the app among adult internet help-seekers.

**Methods:** Adult internet-help seekers, recruited via advertisement, were included if they scored ≥ 6 (women) or ≥ 8 (men) on the Alcohol Use Disorders Identification Test (AUDIT). Those with depression scores of ≥ 31 on the Montgomery Åsberg Depression Rating Scale (MADRS-S) or problematic drug use scores of ≥ 8 on the Drug Use Disorders Identification Test (DUDIT) were contacted for a telephone interview and included following clinical assessment; if not reached, they were excluded. Participants were randomized at a 1:1 ratio to either the TeleCoach™ web-based app or to a web-based app with information texts from primary care-based self-help material for changing problematic alcohol use. At six-week follow-up, the primary outcome was the number of standard drinks per past week (Timeline-Follow back).

**Results:** Of 147 persons assessed for eligibility, 89 were assigned to either the intervention group (n = 42) or control group (n = 47). Average AUDIT levels at baseline were ≥ 18. The baseline number of standard drinks per week was 32.73 (SD 21.16) for the intervention group, and 26 (4.08) for the control group. At 6-week follow-up, the number of standard drinks per week was 12.73 (10.52) and 13.48 (11.13) for the intervention and control groups, respectively. No significant between-groups effects occurred, but within-group changes over time were significant (F = 43.98; p < 0.000), with an effect size of 37 for the intervention group and 2 for the control group.

**Conclusions:** The results suggest that web-based apps can be of help to internet help-seekers who are motivated to reduce problematic alcohol use. Proceeding with a planned larger randomized-controlled study is warranted.

## A4 Web-based therapy versus face-to-face therapy for alcohol dependence

### Magnus Johansson^1^, Kristina Sinadinovic^2^, Ulric Hermansson^2^, Anne H Berman^2^, Sven Andreasson^1^

#### ^1^Department of Public Health, Karolinska Institutet, Stockholm, Sweden; ^2^Centre for Psychiatry Research, Department of Clinical Neuroscience, Karolinska Institutet, & Stockholm Health Care Services, Stockholm County Council, Stockholm, Sweden

##### **Correspondence:** Magnus Johansson - magnus.johansson.1@ki.se

*Addiction Science & Clinical Practice* 2019, **14(Suppl 1):**A4

**Background:** Computer- and internet-based interventions for alcohol problems have been available for over 20 years. Most previous studies have focused on prevention, using screening and brief intervention (eSBI). More extended treatment interventions (iCBT) have been found to reduce alcohol consumption significantly more than control interventions in a small number of studies. There is a need for more studies in a clinical setting, treating people with alcohol use disorder (AUD) and comparisons between internet-based and face-to-face treatment. One reason for using internet-based interventions is lower costs, although only a few economic evaluations have been carried out.

**Methods:** A randomized controlled non-inferiority trial was conducted at a clinic treating AUD. Participants signed up via the clinic website, were assessed by a physician including alcohol biomarkers and were randomized to treatment via internet or face-to-face. The same treatment material and the same psychologists were used in both groups. Follow-up was conducted at 6 months. The non-inferiority limit was set to 5 drinks (60 g of alcohol) a week. Health economic analysis was based on calculation of quality adjusted life years (QALYs) from the EQ-5D.

**Results:** Randomized patients (n = 303) had a mean age of 49 years (SD = 12) and 38% were women. Mean consumption for the previous week was 24 (SD = 14) drinks. Mean AUDIT score was 21 (SD = 5). 66% met criteria for severe AUD. The treatment cost was lower in internet compered to face-to-face treatment. Preliminary analyses show that the weekly alcohol consumption for patients treated face-to-face decreased by 2 more drinks than for those treated via internet (95% CI − 4.5 to 0.5) at 6-month follow-up, indicating non-inferiority for the iCBT treatment compared with face-to-face CBT treatment.

**Conclusions:** Internet-based CBT treatment can be used in specialized care and can be as effective as face-to-face treatment in reducing alcohol use among people with AUD and might be more cost-effective.

**Trial registration:** NCT02888002.

## A5 Prevalence of alcohol misuse problem (AMP) recognition within UK military personnel who meet criteria for alcohol misuse

### Panagiotis Spanakis^1^, Rachael Gribble^2^, Sharon Stevelink^2^, Roberto Rona^2^, Nicola Fear^2^, Laura Goodwin^1^

#### ^1^University of Liverpool, Liverpool, UK; ^2^King’s College London, London, UK

##### **Correspondence:** Panagiotis Spanakis - spanak87@liverpool.ac.uk

*Addiction Science & Clinical Practice* 2019, **14(Suppl 1):**A5

**Background:** Although high rates of alcohol misuse problems (AMP) have been found in the UK military, many military personnel do not reduce their heavy drinking and help seeking is uncommon. Recognition of AMP is a fundamental step in the psychological process of taking action to change behaviour. This study examined data from the most recent wave of a UK military health and well-being cohort study to calculate prevalence of self-identification of existing AMP.

**Methods:** A representative sample of serving and ex-military personnel (n = 8,093) completed a battery of questionnaires, including the Alcohol Use Disorders Identification Test (AUDIT), the General Health Questionnaire (GHQ-12; a measure of common mental health disorders—CMD), and the Post-Traumatic Stress Disorder (PTSD) Checklist (PCL-C). Participants also self-reported whether they have experienced any alcohol problems in the last three years (self-identification of AMP). Data were cross-tabulated to calculate weighted percentages with 95% confidence intervals (CI).

**Results:** 609 participants met criteria for AMP (AUDIT score ≥ 16). Preliminary analyses suggest that 49% (CI = 44%–54%) self-identified their problems. Problems were also self-identified by 8% (7%–9%) of hazardous drinkers (8 ≥ AUDIT < 16), 32% (27%–38%) of harmful drinkers (16 ≥ AUDIT < 20), and 74% (67%–80%) of those with probable dependence (AUDIT ≥ 20). 60% (53%–35%) of those with AMP and CMD comorbidity recognized their AMP, as opposed to 41% (35%–47%) of those with no CMD comorbidity. Also, 68% (57%–78%) of those with AMP and PTSD comorbidity recognized their AMP, as opposed to 45% (40%–50%) of those with no PTSD comorbidity.

**Conclusion:** Around half of UK military personnel do not recognize their AMP. Recognition is higher in more severe cases and in those with comorbid mental health issues. Future studies should focus on policies and interventions that could increase self-awareness of AMP among military personnel.

## A6 Coping with alcohol use disorders: a consumer’s perspective

### Anja Bischof, Miriam Brandes, Tjorven Stamer, Hans-Jürgen Rumpf, Gallus Bischof

#### Department of Psychiatry and Psychotherapy, University of Lübeck, Lübeck, Germany

##### **Correspondence:** Anja Bischof - anja.bischof@uksh.de

*Addiction Science & Clinical Practice* 2019, **14(Suppl 1):**A6

**Background:** The percentage of individuals with alcohol use disorders (AUD) seeking treatment is low. Previous studies have examined barriers to treatment including comorbidity, structural barriers, shame, and stigmatization, mainly based on standardized questionnaires. Nevertheless, the assessment of barriers often does not include in-depth reasons for not seeking treatment.

**Methods:** The study Alcohol-related treatment: a consumer’s perspective (ART-COPE) aims at assessing the development of drinking problems, problem awareness, and coping mechanisms from the perspective of participants using narrative interviews based on Reflective Grounded Theory. Special emphasis lies on the perception of treatment offers in individuals with AUD without treatment experience. Participants with and without treatment experience were recruited pro-actively in general practices and general hospitals. All interviews are recorded, transcribed verbatim, and analyzed using MAXQDA. The study aims to interview 25 individuals. To date, ten interviews have been realized.

**Results:** All individuals reported that drinking was common in familial and social contexts throughout adolescence and adult life. Family was perceived both as a stressor and an accelerator of drinking problems as well as a facilitator for problem awareness and treatment seeking. Alcohol consumption often served as a means for emotion regulation and in some cases as self-medication for depression. Further common topics were loneliness, shame, and stigmatization. Barriers to treatment included fear of losing autonomy and the excessive bureaucratic effort to apply for a therapy. Due to less stable patterns of alcohol dependence, a subgroup reported a sense of control as an additional barrier.

**Conclusions:** To increase the reach for patients with AUD, social structures and families should be strengthened to facilitate access to treatment. Reducing bureaucratic procedures and fostering shared decision making could further increase readiness for treatment. It is expected that all interviews will be analyzed by the date of the conference.

## A7 Brief Intervention for alcohol in pregnant women with criteria for Alcohol Use Disorders: an exploratory study

### Aldana Lichtenberger, Paula V. Gimenez, Raquel Peltzer, Mariana Cremonte

#### Research Group on Psychoactive substances and injuries, Institute of Basic Psychology, Applied and Development of Psychological Technology (IPSIBAT), National Scientific and Technical Research Council (CONICET), National University at Mar del Plata (UNMdP), Buenos Aires, Argentina

##### **Correspondence:** Paula V. Gimenez - gimenezpv@hotmail.com

*Addiction Science & Clinical Practice* 2019, **14(Suppl 1):**A7

**Background:** The sub-population of patients with alcohol use disorders (AUD) is usually excluded from studies on Screening and Brief Intervention (SBI), although SBI might be effective in increasing motivation for behavior change. For that reason, the evidence is scarce among the general population and non-existent for pregnant women. The aim of this exploratory and secondary analysis of data (from an efficacy-randomized study) is to present the results of the acceptability of SBI among a group of pregnant women with criteria for AUD.

**Methods:** 23 pregnant women with criteria for AUDs were identified in a probabilistic sampling of pregnant women who attended the Public Health Centers of Mar del Plata, Argentina, during 2016 (*n *= 893). Every participant received BI and referral to treatment. Screening was performed with the AUDIT (scores 16 were considered positive) and acceptability was assessed with four ad hoc questions. Consumption and related problems were evaluated three months later.

**Results:** Of all the women who were contacted again after three months (*n *= 10), only one increased her AUDIT score, due to the number of standard units consumed per occasion, although episodes of binge drinking decreased. Of the nine participants who decreased their AUDIT scores, seven reported abstinence. All the participants stated that the questions were easy to answer; most of them stated that they learned something new and that they had shared the contents of the interview with others.

**Conclusions:** Despite limitations, these results suggest that SBI may be well accepted among pregnant women with criteria for AUD.

## A8 Understanding Recovery from Alcohol Use Disorders with Systematic Alcohol SBIRT Data

### Constance Weisner^1^, Vanessa A. Palzes^1^, Derek Satre^2^, Stacy A. Sterling^1^

#### ^1^Division of Research, Kaiser Permanente Northern California, Oakland, CA, USA; ^2^Department of Psychiatry, University of California, San Francisco, CA, USA

##### **Correspondence:** Constance Weisner - Constance.Weisner@kp.org

*Addiction Science & Clinical Practice* 2019, **14(Suppl 1):**A8

**Background:** Alcohol use disorders (AUDs) are a health concern and understanding recovery is imperative. Using data from a population-based SBIRT in primary care, we examined correlates of recovery defined as reporting either abstinence or low-risk drinking at a follow-up screening.

**Methods:** We identified 4,078 adults with both an AUD diagnosis and positive screening for unhealthy drinking (any day drinking 5+/4+ and/or 15/8 drinks/week for men/women, respectively) between 10/1/2015 and 9/30/2016, and extracted their electronic health records through 10/2018, allowing a 3-year follow-up. We conducted survival analysis using the Kaplan–Meier method and fit Cox proportional hazards models to examine associations between recovery and patient characteristics, comorbidities, and health service utilization.

**Results:** About 57% of the cohort recovered during our follow-up, with 607 days the median. Controlling for covariates, factors significantly associated with greater odds of having shorter recovery times were female; older age; Black or Latino/Hispanic race/ethnicity; having more medical comorbidities and no drug use disorders; lower drinking severity; and being in addiction treatment within the prior year. Having psychiatric comorbidities in the first year was associated with higher likelihood, while being in psychiatric treatment during that period was associated with lower relative likelihood of recovery at any given time during the follow-up. There was not an association between having a brief intervention for the index positive screening and recovery.

**Conclusion:** In a health system that has implemented systematic SBIRT, we had the unique opportunity to examine correlates of AUD recovery utilizing longitudinal alcohol screening data among individuals with AUD and unhealthy drinking. Our study adds to the growing literature on recovery from a perspective that includes both abstinence and low-risk drinking and suggests that primary care-based SBIRT may help understand the recovery process, including vulnerable subgroups such as those with mental illness, and guard against growing health disparities among ethnic minorities.

## A9 Addiction and post-traumatic stress: evolution of post-traumatic stress symptoms among the Maitre de sa vie program’s participants

### Myriam Laventure, Geneviève Paquette, Jennifer Beauregard

#### Université de Sherbrooke, Sherbrooke, Canada

##### **Correspondence:** Myriam Laventure - Myriam.Laventure@USherbrooke.ca

*Addiction Science & Clinical Practice* 2019, **14(Suppl 1):**A9

**Background:** One person out of four being under treatment for an addiction would present a post-traumatic stress state diagnosis. Individuals experiencing both these situations concomitantly usually draws less benefits from addiction treatments. The addiction relapse rates range from 40% to 60% among the people under treatment for addiction and suffering from post-traumatic stress. The Program Maitre de sa vie—a brief intervention of 6 weekly group workshops—was set up in a Quebec’s addiction rehabilitation center in order to simultaneously target the issues related to addiction as well as to a post-traumatic stress state.

**Method:** The sampling is composed of 75 adults (37 women) having an addiction to alcohol (86.8%) and to drugs (88.1%), and presenting post-traumatic stress symptoms (self-disturbance, post-traumatic stress, exteriorization, somatization). In order to assess the participant’s evolution to the program, data collection including four measurement times was performed: before the program, between the third and fourth meeting, at the end of the program and 3 months after the program. Measurement of post-traumatic stress symptoms was based on regression models including symptoms values when entering the program, gender, age, exposition to the program, therapeutic alliance quality and adaptation strategies used.

**Results and conclusion:** Participation to the Program is associated with a significant decrease of post-traumatic stress symptoms in between each measurement time. A significantly fewer number of participants also reach the clinical thresholds of post-traumatic stress at the end of the program. Among the variables under study, a decrease in post-traumatic symptoms is foreseen while being a male, being younger, having a greatest exposition to the Program, a better quality of therapeutic alliance and using a positive adaptation strategy. The results support the relevance of introducing interventions based on adaptation strategies among the persons presenting both addiction and post-traumatic stress symptoms.

## A10 Predicting Imminent Homelessness Among Emergency Department Patients with Unhealthy Alcohol or Drug Use

### Kelly M. Doran^1^, Eileen Johns^2^, Marybeth Shinn^3^, Maryanne Schretzman^2^, Donna Shelley^1^, Ryan P. McCormack^1^, Lillian Gelberg^4^, John Rotrosen^1^, Tod Mijanovich^5^

#### ^1^NYU School of Medicine, New York City, USA; ^2^NYC CIDI, New York City, USA; ^3^Vanderbilt, Nashville, USA; ^4^UCLA, Los Angeles, USA; ^5^NYU Steinhardt School, New York City, USA

##### **Correspondence:** Kelly Doran - kelly.doran@nyumc.org

*Addiction Science & Clinical Practice* 2019, **14(Suppl 1):**A10

**Background:** Homelessness and substance use often coexist, with each issue exacerbating the other. Both are prevalent among emergency department (ED) patients. Concurrent screening and intervention to prevent homelessness might enhance the effectiveness of ED-based SBIRT.

**Materials and Methods:** We conducted interviews with a random sample of New York City (NYC) public hospital ED patients who screened positive for past year unhealthy alcohol or drug use [using single-item screening questions (Smith et al. 2009, 2010)]. Adult patients were eligible if they spoke English or Spanish, were medically stable, and not in prison/police custody. Using patient identifiers, data were linked to the NYC shelter administrative database, which captures 90% of NYC shelters. The primary outcome was shelter entry within 6 months of the baseline ED visit, among patients who were not already homeless at baseline.

**Results:** Interviews were conducted with 1,262 unique ED patients with unhealthy alcohol or drug use who were not currently homeless. 8.5% had a shelter entry within the next 6 months. Self-judged risk of using a shelter in the next 6 months rated as “somewhat” or “very likely” had 53.3% sensitivity and 26.5% PPV for future shelter entry. A brief homelessness risk screening tool—developed via predictive modeling plus stakeholder feedback—comprising 3 yes/no questions (shelter use in past year, applied for shelter in past 3 months, lifetime incarceration history), with an affirmative answer to any question considered a positive screen, had 85.0% sensitivity and 19.1% PPV.

**Conclusions:** A brief screening tool identified ED patients with unhealthy substance use who were at risk for near-term homeless shelter entry with accuracy similar to screeners developed for other populations, and exceeding the sensitivity of self-assessed risk. If replicated, this screening tool or similar tools could be used to identify which patients with unhealthy substance use may need targeted homelessness prevention services.

## A11 Exploring a Complex Relationship: A Qualitative Study of Substance Use and Homelessness

### Amanda Jurewicz^1^, Deborah Padgett^2^, Ziwei Ran^2^, Donna G. Castelblanco^1^, Ryan P. McCormack^1^, Lillian Gelberg^3^, Donna Shelley^1^, Kelly Doran^1^

#### ^1^NYU School of Medicine, New York City, NY, USA; ^2^NYU Silver School of Social Work, New York City, NY, USA; ^3^University of California, Los Angeles, CA, USA

##### **Correspondence:** Kelly Doran - kelly.doran@nyumc.org

*Addiction Science & Clinical Practice* 2019, **14(Suppl 1):**A11

**Background:** Emergency department (ED) patients commonly face problems with both substance use and homelessness. Research has suggested a bi-directional relationship between substance use and homelessness, but most prior research has been quantitative and cross-sectional. Better understanding this relationship could inform the design of more responsive ED-based substance use interventions, including those that also address homelessness.

**Materials and Methods:** We conducted in-depth, one-on-one interviews with ED patients who had become homeless within the past 6 months. Using a semi-structured interview guide, we asked patients about their pathways into homelessness and the relationship between their substance use and homelessness. Interviews, on average lasting 42 min, were digitally recorded and professionally transcribed. Transcripts were coded line-by-line by 2–3 investigators, who discussed and refined codes in an iterative fashion. The codes then formed the basis for thematic analysis and consensus discussions. ATLAS.ti was used to assist with data organization.

**Results:** Of the 31 patients interviewed, 54.8% reported unhealthy alcohol use and 41.9% drug use in the past year; for others, substance use was only in the past. Five themes emerged: (1) substance use often contributes to homelessness as an upstream factor, through varied intermediary factors (e.g., job loss, family discord); (2) homelessness affects substance use variably, both increasing (e.g., due to depression) and decreasing substance use (e.g., due to lack of time); (3) substance use and homelessness sometimes share precipitants, often related to interpersonal factors; (4) substance use creates practical and environmental barriers relevant to homelessness (e.g., avoiding shelters that might trigger relapse); (5) homelessness can both promote and hinder entry into substance use treatment (e.g., may motivate “change”).

**Conclusions:** Substance use and homelessness are intertwined in complex ways. ED-based substance use interventions should consider the high prevalence of homelessness and the variable ways in which homelessness affects substance use and vice versa.

## A12 Target populations for early interventions in gambling disorder

### Anika Trachte, Dominique Brandt, Anja Bischof, Gallus Bischof, Bettina Besser, Svenja Orlowski, Hannah Hoffmann, Tjorven Stamer, Hans-Jürgen Rumpf

#### Department of Psychiatry and Psychotherapy, University of Lübeck, Lübeck, Germany

##### **Correspondence:** Anika Trachte - Anika.Trachte@uksh.de

*Addiction Science & Clinical Practice* 2019, **14(Suppl 1):**A12

**Background:** Gambling disorder is a rare but serious disease, and most affected individuals do not seek treatment. Especially adolescents and young adults show a high prevalence of subclinical gambling involvement, indicating that early interventions measures might be promising. Aim of this study is to define suitable subgroups in a vocational student setting.

**Methods:** An unselected and non-treatment-seeking sample (n = 6,781) has systematically been screened proactively in vocational schools in Schleswig–Holstein, Germany. Students with at-risk or pathological gambling behavior (n = 1,809) according to the Stinchfield questionnaire were asked to participate in three in-depth telephone interviews, one at baseline, followed by two interviews after approximately 10 and 20 months. A subsample of 405 potential participants were contacted for the baseline interview. Stability of gambling involvement and associated socio-demographic variables were analyzed.

**Results:** The telephone assessments resulted in 309 valid baseline interviews (response rate 78.7%), 268 in the first and 227 in the second follow-up. Of the baseline sample, 43.4% (n = 134) showed at least subclinical gambling involvement (2 or more DSM-5 criteria). Participants with at risk/pathological gambling were significantly more often male (96.3%), had a migration background (72.4%), were single (66.4%), and had a lower school education (88.0%) compared to participants without gambling problems. Regarding the trajectory of gambling involvement, 44.3% reported deterioration of their gambling behavior over time or maintained at least subclinical symptoms from baseline to second follow-up.

**Conclusions:** Students in vocational schools show elevated levels of problematic gambling patterns and can be successfully approached in this setting. Data show that symptoms of pathological gambling are stable in this population and therefore should be addressed using prevention measures. Response rates are comparable to other studies in the field of substance-related Brief Interventions (BI). Implementing BI targeting pathological gambling in vocational schools therefore seems to be a promising strategy.

## A13 Screening and brief interventions for problematic Internet use in adolescents and young adults

### Dominique Brandt, Anja Bischof, Anika Trachte, Bettina Besser, Svenja Orlowski, Hannah Hoffmann, Tjorven Stamer, Gallus Bischof, Hans-Jürgen Rumpf

#### Department of Psychiatry and Psychotherapy, University of Lübeck, Lübeck, Germany

##### **Correspondence:** Dominique Brandt - dominique.brandt@uksh.de

*Addiction Science & Clinical Practice* 2019, **14(Suppl 1):**A13

**Background:** Problematic and pathological Internet use is an important current topic in research and treatment of addictions. Especially adolescents and young adults constitute a vulnerable group of users. Therefore, the effectiveness of brief interventions (BI) to reduce problematic Internet use should be evaluated.

**Method:** Vocational students were screened proactively with the Compulsive Internet Use Scale (CIUS) as well as with other impairment measures. Those with a CIUS score higher than 21 were asked for permission to be approached for a telephone interview. In case that at least two DSM-5 criteria for Internet use disorders were fulfilled during the in-depth interview, participants were randomized into an intervention and a control group. The intervention group received up to three counseling sessions based on Motivational Interviewing. After five and ten months follow-up assessments were conducted.

**Results:** A total of 8,606 students were screened of which more than one-third (n = 3,142) showed problematic Internet usage. This subgroup significantly showed higher impairment in daily tasks and duties. In addition, approximately 80% were concerned to use certain applications too much. Among the 1,481 screening-positive subjects eligible for study participation, 934 interviews could be realized (Response rate 67%). Problematic or pathological Internet use was discovered in 55% (n = 507) of the interviews. In this ongoing study, vocational students’ accessibility via mobile phone proved to be challenging. To realize one BI session several contacts were necessary.

**Conclusion:** Vocational schools are an appropriate setting for offering brief interventions for pathological internet use due to elevated prevalence rates. However, motivation of students to participate in counseling sessions was limited. Brief interventions should be adapted for this target group for example by using smartphone applications.

**Trial registration:** NCT03646448.

## A14 The Sustained Patient-centered Alcohol-Related Care (SPARC) Trial’s Use of Enhanced Practice Coaching to Implement and Sustain Alcohol-related Care in Primary Care

### Amy K. Lee^1^, Carol E. Achtmeyer^2^, Emily C. Williams^3^, Evette J. Ludman^1^, Julie E. Richards^1^, Katharine A. Bradley^1^, Paula Lozano^1^, Rebecca L. Parrish^4^, Ryan M. Caldeiro^4^

#### ^1^Kaiser Permanente Washington Health Research Institute, Kaiser Permanente Washington, Seattle, WA, USA; ^2^Center of Excellence in Substance Abuse Treatment and Education, VA Puget Sound Health Care System, Seattle, WA, USA; ^3^Health Services Research and Development Center of Innovations for Veteran-Centered and Value-Driven Care, VA Puget Sound, Seattle, WA, USA; Department of Health Services, University of Washington, Seattle, WA, USA; ^4^Kaiser Permanente Washington, Seattle, WA, USA

##### **Correspondence:** Amy Lee - amy.k.lee@kp.org

*Addiction Science & Clinical Practice* 2019, **14(Suppl 1):**A14

**Background:** Practice coaches are effective for supporting quality improvement (QI) in primary care (PC). Typically, PC teams possess the clinical expertise, and coaches help teams implement process improvements. But what if PC teams do not have the clinical knowledge needed to improve care? This report presents a model of enhanced practice coaching used in the SPARC trial and findings based on performance metrics.

**Methods:** SPARC was a stepped-wedge pragmatic trial to implement evidence-based care for unhealthy alcohol use in 22 PC clinics. At the request of operations partners, SPARC was rolled out alongside a Behavioral Health Integration initiative. Prior to SPARC, 19% of PC patients completed alcohol screening; there was no standardized assessment for alcohol use disorder (AUD). The intervention had three components: front-line PC support by practice coaches, electronic health record (EHR) tools, and performance feedback. Practice coaches had weekly QI meetings with each clinic’s implementation team for ~ 6 months (“active implementation”). Coaches addressed clinical knowledge gaps, modeled destigmatizing language, and collaborated on EHR tools and performance metrics development. Following active implementation, operations partners continued quarterly QI meetings with PC teams (without coaches). We report findings from performance metrics for all 22 clinics at the end of SPARC (7/2018), and sustainment eight months later (3/2019): % completing alcohol screening among PC patients; and % completing standardized assessment of DSM-5 AUD symptoms among patients with high-risk alcohol screening scores.

**Results:** There were 37,093 PC patient visits across the 22 clinics in 7/2018, and 44,954 in 3/2019. Alcohol screening rates were 88% in 7/2018 and 89% in 3/2019. AUD assessment rates of high-risk patients were 64% in 7/2018, and 70% in 3/2019.

**Conclusion:** Enhanced practice coaching can lead to sustained improvements. Based on sustainment and staff/leader satisfaction, this implementation model has become a “gold standard” for this health system.

## A15 Secondary Evaluation of the Sustained Patient-centered Alcohol-related Care (SPARC) Trial: Patient Reported Advice Across Primary Care Sites at Three Phases of Implementation

### Emily C. Williams^1,2^, Madeline C. Frost^1,2^, Amy K. Lee^3^, Jennifer Bobb^3^, Julie E. Richards^3^, Evette J. Ludman^3^, Carol E. Achtmeyer^1^, Malia M. Oliver^3^, Ryan M. Caldeiro^3^, Rebecca L. Parrish^3^, Joseph E. Glass^3^, Paula Lozano^3^, Katharine A. Bradley^3^

#### ^1^Health Services Research & Development Center for Veteran-Centered and Value-Driven Care, Veterans Affairs Puget Sound Health Care System, Seattle, WA, USA; ^2^Department of Health Services, University of Washington, Seattle, WA, USA; ^3^Kaiser Permanente Washington Health Research Institute, Seattle, WA, USA

##### **Correspondence:** Emily C. Williams - emily.williams3@va.gov

*Addiction Science & Clinical Practice* 2019, **14(Suppl 1):**A15

**Background:** To implement brief intervention for unhealthy alcohol use, SPARC tested state-of-the-art implementation strategies—practice coaching, electronic health record (EHR) decision support, and performance monitoring and feedback—in 22 clinics of Kaiser Permanente Washington (KPW) from 01/15 to 07/18 using a stepped wedge design. Primary results showed that the intervention significantly increased EHR-documented brief intervention, but rates were very low (5%). This presentation uses data from a state-wide patient experience survey conducted in the middle of the SPARC trial to report on and compare rates of patient-reported receipt of brief intervention at sites surveyed before, during or after active implementation.

**Methods:** From 08/17 to 11/17, the Washington Health Alliance survey included questions assessing heavy episodic drinking (HED) and a question assessing receipt of alcohol-related advice (a key component of brief intervention). Sites were categorized into 3 groups, based on their randomly-assigned start date for the SPARC trial: those surveyed before, during or after implementation. For each group of sites, we calculated the percent of surveyed patients who reported alcohol-related advice (“% patient-reported brief intervention”) among those reporting any HED, and compared % patient-reported brief intervention at sites surveyed before, during and after implementation, using Chi-square and test for trend.

**Results:** Five sites were surveyed before SPARC implementation, 3 during and 13 after; % patient-reported brief intervention ranged 13.3% to 55.6% across sites. Rates of patient-reported brief intervention in groups of sites surveyed before, during and after implementation, respectively, were 40.9%, 47.9% and 39.2% (p-values for comparisons all > 0.05).

**Conclusions:** Although rates of patient-reported alcohol-related advice were higher than those based on EHR documentation, no differences in rates of patient-reported brief intervention were observed before during and after SPARC implementation. As in the main trial, results support further quality improvement efforts to ensure patients with unhealthy alcohol use receive brief intervention.

## A16 Preliminary evaluation of a mobile-based Brief Intervention for hazardous drinkers in Goa-India

### Danielle Fernandes^1^, Abhijit Nadkarni^2^, Richard Velleman^3^, Urvita Bhatia^1^

#### ^1^Addictions Research Group, Sangath-Goa, Porvorim, India; ^2^Department of Population Health, The London School of Hygiene and Tropical Medicine, London, United Kingdom; ^3^Department of Psychology, University of Bath, Bath, UK

##### **Correspondence:** Danielle Fernandes - danielle.fernandes@sangath.in

*Addiction Science & Clinical Practice* 2019, **14(Suppl 1):**A16

**Background:** Hazardous drinking (HD) is a major public health problem in India. However, healthcare access is limited by the shortage of healthcare professionals. Extensive global evidence demonstrates the effectiveness of technology-delivered BIs in reducing alcohol consumption. Our study aims to increase healthcare access for HD, by designing a contextually-appropriate mobile-based BI, and evaluating its acceptability, feasibility, and preliminary impact.

**Methods:** Through a systematic review and in-depth interviews with experts and intended recipients, initial content areas for the intervention were derived. These were presented in a Delphi survey to 30 international experts, who rated each area on a five-point Likert scale. At the end of this two-stage iterative process, content areas that reached group consensus were synthesized to inform the intervention development. The draft intervention was then delivered in a case series to participants who screened positive for HD on the Alcohol Use Disorder Identification Test (AUDIT). At one-month follow-up, in-depth interviews were conducted to understand the acceptability and feasibility of the intervention. The preliminary impact was examined through changes in drinking parameters measured using the Timeline-Follow-Back (TLFB).

**Results:** 26 content areas were derived from the systematic review and interviews, and 22 of those met Delphi consensus. The intervention is currently being delivered in the case series, and findings on acceptability, feasibility and impact will be ready for presentation at the conference. Preliminary follow-up interviews (n = 11) have indicated a preference for push messages and an app-based delivery. Higher number of messages was cited as an engagement deterrent, with three messages per week considered ideal.

**Conclusion:** The content and delivery of the intervention will be iteratively refined during the case series, and the final package will be pilot tested through a randomised control trial. If demonstrated to be effective, the intervention will change the landscape of interventions for HD in resource-constrained settings.

## A17 Employment and living arrangement moderate the effectiveness of BI among university students

### Paula V. Gimenez, Karina Conde, Raquel Peltzer, Mariana Cremonte

#### Research Group on Psychoactive substances and injuries, Institute of Basic Psychology, Applied and Development of Psychological Technology (IPSIBAT), National Scientific and Technical Research Council (CONICET), National University at Mar del Plata (UNMdP), Buenos Aires, Argentina

##### **Correspondence:** Paula V. Gimenez - gimenezpv@hotmail.com

*Addiction Science & Clinical Practice* 2019, **14(Suppl 1):**A17

**Background:** Although BI has shown to be effective among university students in high income countries, little research has been done in Latin-America. Furthermore, evidence examining moderators of intervention efficacy is scarce. Certain characteristics that make alcohol more easily available to students, such as living outside of parental control or having economic autonomy to spend money on alcohol, could moderate BI effectiveness. The objective of this study is to evaluate the moderator role of the living arrangements and the employment situation on BI effectiveness.

**Materials and methods:** Participants were 473 students from Mar del Plata National University (60% women, 40% men; between 17 and 46 years old (M = 20.34, SD = 3.9)). Prospective participants were screened and those with high-risk alcohol consumption in the last 12 months were randomly assigned to a control group or BI. After 3 months, they were re-assessed. The measures were: effectiveness (i.e. decrease in AUDIT scores (yes/no)), employment situation (work: yes/no) and living arrangements (living with family: yes/no). Fisher´s exact test was used to analyze the moderator effects of living arrangements and employment situation on effectiveness. Logistic regression analyses were performed in order to control the possible effect of age.

**Results:** 76% of students lived with their families, while 24% lived alone or with friends; 42% of the students were employed. Living with family moderated (increased) BI effectiveness (9,310, p = 0.01). Similarly, not having employment (i.e. being supported by family) also moderated (increased) BI effectiveness (7,611, p = 0.02). These moderator effects were not accounted for by age.

**Conclusions:** Living arrangement and employment moderated effectiveness of BI, suggesting that restricted access to alcohol may improve the effectiveness of interventions among university students.

## A18 Who are the users of the Brazilian self-help intervention program “Bebermenos” (drink less) who accepted to participate in a RCT to evaluate its effectiveness?

### Maria Lucia O. S. Formigoni^1^, André L. M. Andrade^2^, Fabricio Landi-Moraes^3^, Gabrielle A. Cunha^4^

#### ^1^Departamento de Psicobiologia, Universidade Federal de Sao Paulo, Sao Paulo, SP, Brazil; ^2^Faculdade de Psicologia, PUC-Campinas, Campinas, SP, Brazil; ^3^ Departamento de Informática em Saúde, Universidade Federal de São Paulo. Sao Paulo, SP, Brazil; ^4^Departamento de Psicobiologia, Universidade Federal de Sao Paulo, Sao Paulo, Brazil

##### **Correspondence:** Maria Lucia O. S. Formigoni - mlformig@gmail.com

*Addiction Science & Clinical Practice* 2019, **14(Suppl 1):**A18

**Background:** The World Health Organization supported researchers from four countries (Belarus, Brazil, India, and Mexico) in the development of an e-health self-help six-week intervention to reduce alcohol use and related problems. Objective: To describe the profile of users of this web-based intervention.

**Materials and methods:** From September 2016 to January 2019, 579 users who filled out the AUDIT and were considered risk users, or possibly dependent, participated in a Randomized Clinical Trial to evaluate the effectiveness of the intervention. Out of 579, 281 were randomly allocated to the experimental group (virtual Brief Intervention) and 298 to the control group (waiting list). Six months after admission, follow-up was conducted.

**Results:** Of the total sample, most participants (61.1%) were men and 50% were between 33 and 44 years old (median = 36 years). Regarding the classification based on AUDIT scores, 17.8% were risk users (zone II), 16.4% presented harmful/hazardous drinking (zone III) and most (65.8%) were classified as possible dependence users (zone IV). The mean AUDIT total score was 22 (SD = 6.8) and participants reported having consumed about 37 (median) standard drinks in the week prior to entering the program. Regarding the Readiness to Change questionnaire scores, most participants were classified in the contemplation phase.

**Discussion:** Although designed for at-risk users, most of the users who registered on the site already had severe alcohol-related problems. These data suggest there is a hidden population that should be under treatment for alcohol dependence, but refuse to do it, do not look for it or even do not find available treatment. Internet interventions could help these people by raising awareness of their alcohol-related problems and encourage them to enter the action phase and look for treatment.

**Trial registration:** ISRCTN14037475.

## A19 Increasing access to treatment services for alcohol and substance service users in low income countries: lessons learnt from a trial brief intervention program in Uganda

### Sylvia T. Nabirye, David Kalema

#### Hope and Beyond, Kampala, Uganda

##### **Correspondence:** Sylvia T. Nabirye - nabiryesylvie@yahoo.com

*Addiction Science & Clinical Practice* 2019, **14(Suppl 1):**A19

**Background:** Low income countries are faced with a growing challenge of negative alcohol and substance use, yet interventions are scarce and rarely documented. Uganda is estimated to have 3,900,000 people with alcohol use disorders, yet the country’s main source of treatment for addictive disorders is the National Mental Referral Hospital situated in the capital city. The aim of this paper is to highlight brief intervention as an alternative treatment for Alcohol and Substance Use Disorders (SUD), the likely challenges and potential solutions for this strategy in a low resource setting.

**Methods:** As a way of evolving culturally appropriate services, Hope and Beyond conducted a 5 days’ residential camp to treat and sensitize communities about SUD. The pilot treatment camp was held at Kisigula Health Centre (HC) II in Wakiso District; a metropolitan area that houses many city dwellers and nationals from Uganda and surrounding countries. Residents in the program catchment area were also mobilized to contribute towards logistical needs of camp participants. Camp activities included screening and assessments, detoxification and medications; psychotherapies; HIV counseling and testing, sensitization workshops, prayers/spiritual support and referrals. Challenges faced ranged from logistical to human resource constraints, yet many clients were in severe physical and mental condition and low on motivation.

**Results:** Although the project was planned for 20 participants, 53 clients turned up and were treated for SUD, 12 health workers from nearby medical centers were trained in addiction management and sensitization was conducted in 11 Churches and 2 Mosques reaching out to over 10,000 people.

**Conclusion:** Camp treatment as a way of brief intervention for alcohol and substance use disorders is a promising practice for alternative SUD treatment that should be adapted in low income countries but scientific studies are necessary to establish its effectiveness.

## A20 Clinical needs of participants in brief intervention treatment for alcohol and other substance use in Kampala, Uganda

### David Kalema

#### Hope and Beyond, Kampala, Uganda

##### **Correspondence:** David Kalema - kalemdav@yahoo.com

*Addiction Science & Clinical Practice* 2019, **14(Suppl 1):**A20

**Background:** Brief intervention is promoted as a cost-effective measure to reduce alcohol consumption and prevent alcohol-related harm. However, there is scant evidence on its implementation in developing countries. This paper offers insight into the clinical treatment needs of alcohol and other substance users in low income countries by reporting the prevalence of alcohol and drug use disorders and the co-occurring illnesses amongst residential treatment participants in Kampala, Uganda.

**Methods:** 53 participants (50 males and 3 females) reporting for a 5 day residential treatment camp were interviewed regarding their perception of their physical and mental wellbeing, and screened for drug use disorder(s) using the Alcohol and Substance use Screening Involvement Tool. The treatment camp was held in the Wakiso District near the capital city of Uganda, Kampala.

**Results:** Alcohol was the most commonly consumed drug, used by 66% of participants, followed by nicotine and cannabis at 23% and 8% respectively. 43% were diagnosed with a single substance use disorder and the remainder had multiple use disorders, of which, 36% reported addiction to two drugs and 18.3% used three or more drugs. 86% reported co-occurring physical medical conditions such as fever, sexually transmitted diseases (STI) and/or a cough; 40% reported psychiatric symptoms such as psychosis, insomnia and bipolar; and 57% reported psychological symptoms such as depression and anxiety.

**Conclusion:** Participants of brief intervention treatment for alcohol and substance use in low income settings have varying needs resulting from multiple drug use disorders and other co-occurring medical, psychiatric and psychological illnesses. Brief interventions for alcohol and substance use disorders delivered in free treatment camps should include a range of additional services to meet the participants’ varying multiple and complex needs. Further research is necessary to establish culturally sensitive effective treatment approaches and modalities of brief interventions in developing countries.

## A21 Impact of health disparities and brief counseling interventions on drinking outcomes in hospitalized Trauma patients

### Elizabeth B. White^1^, Emily Wall^2^, Elizabeth Shilling^3^, Laura Veach^3^, Mary C. O’Brien^3^, Preston Miller^3^

#### ^1^Wake Forest Baptist Medical Center, Winston Salem, NC, USA; ^2^Wake Forest Graduate School of Arts & Sciences, Winston Salem, NC, USA; ^3^Wake Forest School of Medicine, Winston Salem, NC, USA

##### **Correspondence:** Emily Wall - emwall@wakehealth.edu

*Addiction Science & Clinical Practice* 2019, **14(Suppl 1):**A21

**Background:** Alcohol use has been shown to increase an individual’s likelihood to experience a traumatic injury, particularly those who misuse, use at risky levels, or have a use disorder. Based on this information, screening and brief counseling interventions (BCIs) can be particularly impactful in hospital trauma center settings to address these patterns. Recently, efforts have grown to understand health disparities in order to provide the best treatment possible. This study aimed to understand how health disparity related factors, namely age, geographic location, insurance type, and sex, as well as two distinct BCIs uniquely predict changes in drinking patterns.

**Methods:** This study was a retrospective analysis on a pre-existing dataset collected from hospitalized trauma patients to evaluate two different BCIs for patients with alcohol-related injuries. The initial study found that both quantitative BCIs and personalized BCIs were effective in reducing self-reported drinking patterns at a six-month follow up with the AUDIT screening tool. This retrospective analysis sought to add depth to these findings by understanding the impact that the aforementioned factors have on predicting these same drinking patterns. After statistically controlling for other variables, each unique health disparity factor and intervention type was tested through hierarchical regressions to determine its contribution.

**Results:** Results demonstrated that regardless of health disparity factors or BCI type, on average, patients displayed reduction in drinking patterns at the six-month follow up. These results also indicated that though all patients experienced improvements, females were more likely to show greater changes than men in either intervention.

**Conclusion:** Overall, this study supports and further highlights the evidence that the use of BCIs containing more innovative and empathy-based approaches are appropriate for impacting positive patient change behaviors. In addition, these study results show that this more flexible approach is appropriate in sub-populations that are more likely to experience health disparity.

## A22 Addiction and Suicide Interventions Specialized for Trauma (ASIST) patients

### Elizabeth H. Shilling, Laura J. Veach, Olivia H. Smith, Elizabeth B. White

#### Department of Surgery, Wake Forest Baptist Health, Winston Salem, NC, USA

##### **Correspondence:** Olivia H. Smith - ocurrin@wakehealth.edu

*Addiction Science & Clinical Practice* 2019, **14(Suppl 1):**A22

**Background:** In 2010, over 650,000 hospital visits were related to attempted suicide. Risk factors for suicide include substance use disorders, mental health disorders, and other demographic and sociocultural factors (Hawton et al. 2013). Addressing both substance use and suicidal ideation is critical in integrated care settings, as those who have been previously hospitalized following suicide attempts are at significantly greater risk of subsequent attempts and hospitalizations (O’Connor et al. 2015). The aim of this preliminary study is to demonstrate the results of providing Screening and Brief Interventions (SBI) for patients who have suicidal ideation (SI) or were hospitalized due to a suicide attempt.

**Materials and Methods:** This preliminary study looked at the feasibility of SBI for substance use and suicide in a Level-1 trauma center. A sample of patients were identified based on hospitalization due to suicide attempt or SI comorbidity with substance use, and were placed in either a control group or intervention group to receive a specialized SBI. Hospital readmission rates were measured to identify the impact of these interventions.

**Results:** Of the intervention group, none of the patients were readmitted to the hospital within thirty days due to repeated suicide attempts or SI. In contrast, within the control group, 37.5% were readmitted within thirty days of their initial hospitalization, of which 25% were hospitalizations related to additional suicide attempts or SI.

**Conclusions:** Preliminary findings support a positive trend: patients that received specialized SBIs addressing both substance use and SI show a reduction in hospital readmissions. Given these findings, further research is warranted regarding the effectiveness of specialized SBIs in patients who have attempted suicide or active SI.

## A23 Counselor-provided SBIRT for hospitalized adults with substance misuse or disordered use: evaluating hospital utilization outcomes

### Marcia H. McCall

#### Wake Forest School of Medicine, Winston Salem, NC, USA

##### **Correspondence:** Marcia H. McCall - mmccall@wakehealth.edu

*Addiction Science & Clinical Practice* 2019, **14(Suppl 1):**A23

**Background:** This study’s purpose was to determine whether SBIRT interventions by professional counselors working on inpatient integrated care settings are effective treatments for alcohol and illicit drug misuse and disordered use. Effectiveness was determined by evaluating the association between interventions and subsequent hospitalizations and emergency department visits. Inpatient settings were selected for study given inconclusive results from prior research for this category of SBIRT recipient. This study controlled for type (alcohol, illicit drugs, or both) and severity of substance use, with inpatient clinical service as a clustering variable.

**Materials and methods:** Using a difference-in-differences approach and generalized linear mixed modeling, 1,577 hospitalized patients receiving SBIRT interventions were compared to 618 patients identified for but not receiving interventions, for a single U.S. hospital over a four-year period. Utilization data were collected one year prior to and following the identifying hospitalization, along with substance use type and severity and clinical service. Propensity scores were developed from demographic, disease, and insurance indicators and used as covariates.

**Results:** On average, patients receiving counselor-provided SBIRT interventions experienced 22% fewer subsequent hospitalizations and emergency department visits than patients not receiving interventions, controlling for substance use type and severity. Outcomes varied significantly across inpatient clinical services. The study sample was 74% male and 73% White, with a mean age of 44.7 years.

**Conclusions:** The study tested a novel substance use treatment model, counselor-provided SBIRT, for a population with a wide spectrum of substance use types and levels of severity. The results offer support for this process as an effective treatment model for reducing utilization of hospitalizations and emergency department visits. Given these findings, health system administrators, physicians, and community leaders may support integrating professional counselors into hospital units and other medical settings, raising the likelihood that people who need help with their substance use actually receive it.

## A24 Scaling up a healthcare workforce to deliver Screening, Brief Intervention and Referral to Treatment (SBIRT): a pilot project

### Yovan Gonzalez, Sharon L. Kozachik, Deborah S. Finnell

#### Johns Hopkins School of Nursing, Baltimore, MD, USA

##### **Correspondence:** Yovan Gonzalez - ygonzal2@jhu.edu

*Addiction Science & Clinical Practice* 2019, **14(Suppl 1):**A24

**Background:** As the largest profession in the healthcare workforce, nurses have important roles in moving evidence related to Screening, Brief Intervention, and Referral to Treatment (SBIRT) to action to address the global burden associated with alcohol and other drug (AOD) use. The purpose of this quality improvement project was to evaluate an online self-paced educational program, Screening, Brief Intervention, and Referral to Treatment (SBIRT) for Healthcare Providers among nurses in an ambulatory care facility with the goal of increasing their knowledge in the screening and management of patients with AOD use.

**Methods:** A one-sample, pretest–posttest design was used in this project. The Wilcoxon Signed Rank Test was used to analyze results from the SBIRT-related knowledge test from before to after the intervention. Descriptive statistics were used to analyze data related to previous SBIRT education and confidence to deliver SBIRT in practice. Thematic analysis was used to categorize barriers to and facilitators for SBIRT implementation.

**Results:** There was a significant increase in SBIRT-related knowledge (p < .001) from before to after the intervention. A high proportion of the nurses had no SBIRT knowledge (45%) prior to the intervention. Nurses reported high confidence levels to screen for alcohol and drugs after the intervention. Barriers to and facilitators for SBIRT implementation related to five themes: (1) time, (2) education, (3) resources, (4) receptivity and (5) interprofessional collaboration.

**Conclusion:** It was feasible to deliver this online SBIRT education to nurses at a busy ambulatory care facility and impactful in terms of increasing SBIRT-related knowledge and confidence. To promote system-wide readiness for widescale dissemination, providing this online program to other ambulatory care clinics and other healthcare professionals is warranted.

## A25 It’s not just what you do, it’s how you do it: variation in substance use screening outcomes with commonly used screening approaches in primary care clinics

### Jennifer McNeely^1^, Joseph L. Kannry^2^, Richard N. Rosenthal^3^, Sarah E. Wakeman^4^, Timothy E. Wilens^4^, Sarah Farkas^5^, Angeline Adam^5^, Carmen L. Rosa^6^, Aimee Wahle^7^, Seth Pitts^7^, John Rotrosen^5^

#### ^1^Dept. of Population Health, NYU School of Medicine, New York, NY USA; ^2^Icahn School of Medicine at Mt. Sinai, New York, NY, USA; ^3^Stony Brook University Medical Center, Stony Brook, NY, USA; ^4^Massachusetts General Hospital, Boston, MA, USA; ^5^NYU School of Medicine, New York, NY USA; ^6^National Institute on Drug Abuse, Center for the Clinical Trials Network, Bethesda, MD USA; ^7^The Emmes Company, Rockville, MD, USA

##### **Correspondence:** Jennifer McNeely - jennifer.mcneely@nyulangone.org

*Addiction Science & Clinical Practice* 2019, **14(Suppl 1):**A25

**Background:** Primary care clinics often struggle to choose the approach to alcohol and drug screening that is best suited to their resources, workflows, and patient populations. We are conducting a multi-site study to inform the implementation and feasibility of electronic health record (EHR)-integrated screening.

**Methods:** In two urban academic health systems, researchers worked with stakeholders from 6 clinics to define and implement their optimal screening approach. All clinics used single-item screening questions for alcohol/drugs followed by AUDIT-C/DAST-10. Clinics chose between: (1) screening at routine vs. annual visits; and (2) staff-administered vs. computer self-administered screening. Results were recorded in the EHR, and data was extracted quarterly to describe implementation outcomes including screening rate and detected prevalence of unhealthy (moderate-high risk) use among those screened. Findings are from the first 3–12 months post-implementation at each clinic.

**Results:** Across sites, of 84,311 patients with primary care visits, 58,492 (69%) were screened. In the 4 clinics with mature (9–12 months) implementation, screening rates ranged from 42 to 95%. Rates were lower (10–22%) in the 2 clinics that recently launched. Screening at routine encounters, in comparison to annual visits, achieved higher screening rates for alcohol (90–95% vs. 42–62%) and drugs (90–94% vs 38–60%). Staff-administered screening, in comparison to patient self-administered screening, had lower rates of detection of unhealthy alcohol use (2% vs. 15–37%). Detection of unhealthy drug use was low, ranging from 0.3 to 1.5%.

**Conclusions:** EHR-integrated screening was feasible to implement in at least 4 of the 6 clinics; 1-year results (available Fall 2019) will determine feasibility at all sites. Self-administered screening at routine primary care visits achieved the highest rates of screening and detection of unhealthy alcohol use. Although limited by differences among clinics and their patient populations, this study provides insight into outcomes that may be expected with commonly used screening strategies in primary care.

**ClinicalTrials.gov identifier:** NCT02963948.

## A26 Dissemination of a web-based program to reduce drug use in Mexico, are we ready to implement?

### Marcela Tiburcio^1^, Nora Martínez-Vélez^1^, Morise Fernández^1^, Ma. Asunción Lara^2^

#### ^1^Department of Social Sciences in Health, Instituto Nacional de Psiquiatría Ramón de la Fuente Muñiz, Mexico City, Mexico; ^2^Department of Innovation and Global Health, Instituto Nacional de Psiquiatría Ramón de la Fuente Muñiz, Mexico City, Mexico

##### **Correspondence:** Marcela Tiburcio - tibsam@imp.edu.mx

*Addiction Science & Clinical Practice* 2019, **14(Suppl 1):**A26

**Background:** Web-based programs for substance use have been designed, evaluated and implemented over the past two decades in high-income countries. The development of such tools in Latin America is more recent. One of the few available web-based programs is the Programa de Ayuda para Abuso de Drogas y Depresión (PAADD). Its feasibility was demonstrated through a randomized trial. The next step is to design a strategy to promote its implementation.

**Methods:** This study aimed at identifying the factors involved in the implementation of technological innovation in Mexico. The level of readiness to adopt technologies for health-care provision was measured with an adapted version of the Telehealth Capacity Assessment Tool (TCAT), which considers 6 domains: organizational, technology, regulatory, financial, clinical, and workforce factors. Free training was offered at 12 substance use prevention and treatment institutions. The managers were asked to complete the TCAT before the training.

**Results:** We received eight completed questionnaires: four from treatment centers affiliated to Psychology Schools in two Universities; one from an immune-infectious clinic, and three from Primary Care Centers for Addictions (PCCA). Additionally, professionals who were trained provided information about: internet use; academic background; experience in substance use treatment and attitudes towards the use of technology. The highest TCAT scores (3–4.5) were observed at the clinic, showing a high degree of readiness to implement web-based programs, the lowest scores (0–2.5) belong to the PCCA, where the implementation is challenging.

**Conclusions:** The information provided by the professionals indicates a negative attitude towards technology and less success in enrolling clients in web-based program at institutions with a low TCAT score, while professionals at institutions with moderate scores were more successful at enrolling and had positive attitudes. The data is relevant to create a dissemination strategy to approach the misconceptions about web-based interventions and facilitate its acceptance as a valid therapeutic alternative.

## A27 Proactive computer-based interventions simultaneously targeting hazardous alcohol consumption and depressiveness: preliminary findings from a randomized controlled proof of concept trial

### Christian Meyer^1,2^, Kristian Krause^1^, Diana Guertler^1,2^, Anne Moehring^1,2^, Jennis Freyer-Adam^2,3^, Sophie Baumann^2,3,4^, Sabina Ulbricht^1,2^, Anil Batra^5^, Sandra Eck^5^, Gallus Bischof^6^, Hans-Jürgen Rumpf^6^, Ulrich John^1,2^

#### ^1^Institute of Social Medicine and Prevention, University Medicine Greifswald, Greifswald, Germany; ^2^DZHK (German Center for Cardiovascular Research), Partner Site Greifswald; Greifswald, Germany; ^3^Institute for Medical Psychology, University Medicine Greifswald, Greifswald, Germany; ^4^Institute and Policlinic for Occupational and Social Medicine, Technische Universität Dresden, Dresden, Germany; ^5^Department of Psychiatry and Psychotherapy, University Hospital of Tübingen, Tübingen, Germany; ^6^Department of Psychiatry and Psychotherapy, University of Lübeck, Lübeck, Germany

##### **Correspondence:** Christian Meyer - chmeyer@uni-greifswald.de

*Addiction Science & Clinical Practice* 2019, **14(Suppl 1):**A27

**Background:** We developed a fully automatized computer-based intervention to address alcohol consumption and depression simultaneously. In the present paper, we report an initial proof of concept trial.

**Methods:** Participants were recruited via a multicenter screening program approaching adult patients from ambulatory practices and hospitals. Inclusion criteria were hazardous alcohol consumption and an episode of subclinical or clinical symptoms of depression in the past year. Patients with current severe depression or indication of alcohol dependence were excluded. In total, 132 participants were randomized to an assessment only control or an intervention group receiving six individually tailored motivational feedback letters and weekly text messages over a period of 6 months. Intervention content was constructed based on the principals of the Transtheoretical Model of behavior change. Outcome was assessed by computer-assisted telephone interviews scheduled 6, 12 and 24 months after baseline.

**Results:** Preliminary analyses were based on data from 6- (n = 104) and 12-month (not completed, current state of April 2019: n = 107) follow-ups. Generalized estimating equation analysis adjusting for recruitment setting, age, and sex revealed a significant decrease in depression scores (p < .01) and no significant time effect for alcohol measures. After 12 months, changes in alcohol and depression measures were numerically larger in the intervention compared to the control group, with small to medium effect-sizes (Cohen’s d: heavy drinking days = 0.36, mean daily consumption = 0.25, depression score = 0.29), but statistical significance was only reached for frequency of heavy drinking days (t-test, one-sided p = .03)

**Conclusions:** The intervention and research logistic proved to be technically feasible. Based on our preliminary analysis, effects seem comparable to single focused motivational interventions among unselected samples. Thus, a future adequately powered effectiveness trial is warranted. Given the low baseline motivation to adopt healthy behaviors final conclusion on effectiveness should be postponed to the availability of long-term outcome data.

**Trial registration:** German Clinical Trials Register DRKS00011635.

## A28 How severity affects short-term effects of a computer-based brief intervention addressing the full spectrum of alcohol use: results from a randomized controlled trial

### Sophie Baumann^1^, Andreas Staudt^2^, Jennis Freyer-Adam^3^, Gallus Bischof^4^, Christian Meyer^1^, Ulrich John^1^

#### ^1^Institute and Policlinic of Occupational and Social Medicine, Faculty of Medicine, Technische Universität Dresden, Dresden, Germany; ^2^Institute of Social Medicine and Prevention, University Medicine Greifswald, Greifswald, Germany; ^3^Institute for Medical Psychology, University Medicine Greifswald, Greifswald, Germany; ^4^Department of Psychiatry and Psychotherapy, University Lübeck, Lübeck, Germany

##### **Correspondence:** Sophie Baumann - sophie.baumann@mailbox.tu-dresden.de

*Addiction Science & Clinical Practice* 2019, **14(Suppl 1):**A28

**Background:** The public health impact of brief alcohol interventions (BAIs) might be increased by approaching an entire population rather than selected high-risk individuals only. In this study, all persons who drink alcohol were offered BAI, including those identified as having low-risk alcohol use or with greater severity. The aim was to investigate the BAI efficacy during the active intervention phase as a function of alcohol use severity.

**Methods:** In our ongoing randomized controlled trial (http://www.drks.de/DRKS00014274), we systematically screened all persons aged 18–64 years appearing in the waiting area of a local registration office over a period of two months. Those who reported alcohol use in the past 12 months (n = 1,648) were randomized to BAI or assessment only. BAI consisted of computer-generated individualized feedback letters delivered at baseline, month 3, and month 6. Latent growth modeling was used to test BAI effects through the 6 months of intervention as a function of the Alcohol Use Disorders Identification Test-Consumption (AUDIT-C) score. By the cut-off date for this analysis, 6-month assessments have been completed and two of three interventions have been delivered.

**Results:** The trial participation rate was 67%. Three- and 6-month retention rates were 85% and 81%, respectively. Participants with lower AUDIT-C scores were more likely to participate in the trial (OR = 1.07, p = 0.010) and in multiple BAIs (OR = 1.11, p = 0.003) than those scoring high on the AUDIT-C. At month 6, BAI produced significant changes in the number of drinks per week among participants with low AUDIT-C scores (IRR = 0.83, p = 0.035). Effects decreased with increasing AUDIT-C scores (IRR = 1.04, p = 0.048).

**Conclusions:** We provided a computer-based BAI that may be particularly appropriate for the large but understudied group of persons with low severity. Twelve-month data need to be included in analyses before we can draw more definite conclusions about its efficacy in the population as a whole.

**Trial registration:** German Clinical Trials Register DRKS00014274.

## A29 Screening for alcohol use disorder in the general population: an empirical investigation of evidence-based assessments

### Stéphanie Baggio^1^, Bastien Trächsel^2^, Valentin Rousson^2^, Frank Sporkert^3^, Jean-Bernard Daeppen^4^, Gerhard Gmel^4^, Katia Iglesias^5^

#### ^1^Division of Prison Health, Geneva University Hospitals, Geneva, Switzerland; ^2^Division of Biostatistics, Center for Primary Care and Public Health, University of Lausanne, Lausanne, Switzerland; ^3^Centre of Legal Medicine, Forensic Toxicology and Chemistry Unit, Lausanne and Geneva Universities, Lausanne, Switzerland; ^4^Addiction Medicine, Department of Psychiatry, Lausanne University Hospital, Lausanne, Switzerland; ^5^School of Health Sciences, HES-SO University of Applied Sciences and Arts of Western Switzerland, Fribourg, Switzerland

##### **Correspondence:** Stéphanie Baggio - stephanie.baggio@hcuge.ch

*Addiction Science & Clinical Practice* 2019, **14(Suppl 1):**A29

**Background:** Population-based screening of alcohol use disorder (AUD) are crucially needed for public health planning. Evidence-based measurements are needed, but empirical studies comparing different self-reported measures to a gold standard are scarce. This study aimed at identifying a valid screening tool.

**Methods:** This Swiss controlled study collected data among young men from the ongoing Cohort Study on Substance Use and Risk Factors, using a stratified random sample selection (n = 233). AUD was diagnosed using the Diagnostic Interview for Genetic Studies (gold standard). Self-reported measures included criteria of AUD, alcohol-related consequences, and previous twelve-month alcohol use. We tested psychometric performances of the self-reported measures deriving sensitivity and specificity from receiver operating characteristics curves and using all possible subsets of questions.

**Results:** Taken separately, none of the self-reported measures displayed good psychometric properties, maximizing sensitivity and specificity. This was true for the self-reported AUD (cut-off of two or more symptoms: sensitivity = 92.3%, specificity = 45.8%; cut-off of four or more symptoms: sensitivity = 60.3%, specificity = 87.1%) and alcohol use (cut-off of 10 drinks per week: sensitivity = 85.9%, specificity = 55.5%; cut-off of 21 drinks per week: sensitivity = 38.5%, specificity = 93.6%). The best model combined 8 self-reported AUD criteria and 4 alcohol-related consequences. With a cut-off of 3, this screening tool displayed good sensitivity (83.3%) and specificity (78.7%).

**Conclusions:** These findings provided important insights among young men in current debate in the alcohol field: heavy alcohol use was not a suitable single criterion to assess AUD and consequences were important to identify a valid assessment. Even if alcohol use is not part of the final screening tool, it should not be neglected, as it is responsible of a large burden of disease and detrimental health consequences.

## A30 Assessing heavy alcohol use and risky single occasion drinking using an alcohol biomarker among young Swiss men

### Katia Iglesias^1^, Frank Sporkert^2^, Jean-Bernard Daeppen^3^, Gerhard Gmel^3,4,5,6^, Stéphanie Baggio^7,8^

#### ^1^School of Health Sciences, HES-SO University of Applied Sciences and Arts of Western Switzerland, Fribourg, Switzerland; ^2^Centre of Legal Medicine, Forensic Toxicology and Chemistry Unit, Lausanne and Geneva Universities, Lausanne, Vaud, Switzerland; ^3^Addiction Medicine, Department of Psychiatry, Lausanne University Hospital, Lausanne, Vaud, Switzerland; ^4^Addiction Switzerland, Lausanne, Vaud, Switzerland; ^5^Centre for Addiction and Mental Health, Toronto, ON, Canada; ^6^University of the West of England, Bristol, UK; ^7^Division of Prison Health, Geneva University Hospitals and University of Geneva, Thônex, Geneva, Switzerland; ^8^Department of Forensic Psychiatry, Institute of Forensic Medicine, University of Bern, Bern, Switzerland

##### **Correspondence:** Katia Iglesias - Katia.Iglesias@hefr.ch

*Addiction Science & Clinical Practice* 2019, **14(Suppl 1):**A30

**Background:** Empirical studies on the quality of self-reported alcohol use as measures of excessive drinking compared to objective measures, such as ethyl glucuronide (EtG), are needed. In addition, associations of EtG with risky single occasion drinking (RSOD, i.e., ≥ 6 drinks on a single occasion) have been scarcely investigated. This study tested whether self-reported measures of alcohol use and RSOD allow detecting excessive chronic drinking as assessed by EtG.

**Methods:** Data were collected among young Swiss men, recruited in the ongoing Cohort Study on Substance Use and Risk Factors using a stratified random selection. Assessments included self-reported measures of alcohol use (previous twelve-month and previous-week) and RSOD. Capillary blood was collected to determine EtG (n = 227). Data were analyzed using receiver operating characteristics curves, using EtG (cut-off of 30 pg/mg) as the gold standard of excessive drinking. Sensitivity and specificity were computed. We also performed a multivariate logistic regression to test whether alcohol use and RSOD were uniquely associated with EtG.

**Results:** Overall, 23.4% of the participants presented a chronic excessive drinking according to the EtG cut-off of 30 pg/mg. For previous twelve-month alcohol use, a cut-off > 15 drinks per week yielded acceptable psychometric performance (sensitivity = 75.5%, specificity = 78.7%). No cut-off maximized sensitivity and specificity for previous-week alcohol use. Weekly RSOD detected EtG with acceptable psychometric properties (sensitivity = 75.5%, specificity = 70.1%). Sensitivity and specificity were respectively maximized for monthly RSOD (sensitivity = 94.3%) and daily RSOD (specificity = 98.9%). In the multivariate logistic regression, both previous twelve-month alcohol use with a cut-off of 15 and weekly RSOD were significantly associated with EtG (respectively p < .001 and p = .022).

**Conclusion:** Self-reported measures of RSOD and of previous twelve-month alcohol use were acceptable measures of excessive drinking for population-based screening. Self-reported RSOD appeared as an interesting screening measure to identify accurately excessive drinking among young people.

## A31 Development of the COS for health economic research on alcohol brief interventions

### Jeremy Bray^1^, Carolina Barbosa^2^

#### ^1^University of North Carolina at Greensboro, Greensboro, NC, USA; ^2^RTI International, Chicago, IL, USA

##### **Correspondence:** Jeremy Bray - JWBRAY@uncg.edu

*Addiction Science & Clinical Practice* 2019, **14(Suppl 1):**A31

**Background:** Systematic reviews suggest that cost-effectiveness evidence for ABI in emergency care and hospital settings is scarce and that the cost-effectiveness evidence for primary care is not based on a consistent set of economic outcomes. These reviews, as well as future economic evaluations of ABI, are hampered by the lack of a consensus on what economic outcomes should be measured in ABI evaluations. In this presentation, we present a preliminary methodology to establish a core outcome set (COS) for economic evaluations of ABI. This COS is intended to be supplemental to the COS developed for ABI trials by the INEBRIA Research Measurement Standardization Special Interest Group (RMS-SIG).

**Methods:** We present a rapid review of the ABI economic evaluation literature as a first step towards developing an ABI economic COS. Our review began by first mining the existing Outcome Reporting in Brief Intervention Trials: Alcohol (ORBITAL) systematic review database to assess outcomes use in previous economic evaluations of ABI trials. We then supplemented the ORBITAL review with a rapid review specifically designed to identify any gaps in our literature database.

**Results:** We find that ABI economic evaluations seldom use consistent measures, but an increasing number of studies report quality adjusted life years (QALYs) in addition to measures of social costs. Studies suggest that the RMS-SIG should consider measures of: health state utility as derived from health-related quality of life; health care use; injuries and accidents, including motor vehicle accidents; crime and criminal justice involvement; employment, workplace productivity, and absenteeism; and, for adolescent studies, educational outcomes such as school attendance and matriculation.

**Conclusion:** To support the development of a rigorous evidence base for the economic benefits of ABI, the RMS-SIG should develop a core set of economic outcome measures that build on the ORBITAL COS.

## A32 Development of a COS for implementation studies on alcohol brief interventions

### Amy J. O’Donnell^1^, Anne H. Berman^2^, Zarnie Khadjesari^3^

#### ^1^Newcastle University, Newcastle upon Tyne, UK; ^2^Karolinska Institutet, Center for Psychiatry Research, Stockholm, Sweden; ^3^University of East Anglia, Norwich, UK

##### **Correspondence:** Amy J. O’Donnell - amy.odonnell@newcastle.ac.uk

*Addiction Science & Clinical Practice* 2019, **14(Suppl 1):**A32

**Background:** With robust evidence for effectiveness, recent alcohol screening and brief intervention (ASBI) studies have focused on the challenge of encouraging their implementation in routine healthcare. However, as in other intervention fields, outcomes to assess ASBI implementation are currently defined in different ways, and assessed by different measures. Under the INEBRIA Research Measurement Standardization Special Interest Group (RMS-SIG), progress has been made to establish a core outcome set to assess ASBI effectiveness and efficacy. However there remains a need to identify which implementation outcome measures are most appropriate for this field. This presentation will identify which outcomes and associated measurement instruments are currently employed in implementation-focused research overall, and consider their potential applicability to ASBI.

**Methods:** We scrutinised existing systematic reviews (Proctor 2011; Lewis 2015; Khadjesari unpublished) to identify outcomes and associated measurement instruments employed in implementation-focused research. We also searched databases of outcomes assessed in the existing ASBI trial literature (Shorter et al. in press) to determine whether any appropriate implementation outcomes/measures were included.

**Results:** Proctor’s taxonomy identifies a core set of implementation outcomes (acceptability; adoption; appropriateness; cost; feasibility; fidelity; penetration; sustainability). However whilst previous research has employed various implementation outcome measures with relevance to mental, behavioural and/or physical health (n = 154 studies), most assess intervention acceptability (n = 77) and/or adoption (n = 27), and are of relatively low psychometric quality. There is a particular need to develop instruments to assess feasibility, appropriateness and sustainability. Current ASBI trials do not collect implementation outcome data or employ relevant measurement instruments when assessing effectiveness.

**Conclusions:** Recognised outcome taxonomies exist to support efforts to improve the quality and consistency of ASBI implementation research. However, there is an identified lack of robust instruments to support their measurement. Future research is needed to evaluate the status of ASBI implementation research, and to develop/validate instruments relevant to the field.

## A33 Predictive models for the emergence of alcohol or other drug use problems during ages 12–18: opportunities for targeting screening and brief interventions

### Stacy Sterling^1^, Stacey Alexeeff^1^, Beth Waitzfelder^2^, Brian K. Ahmedani^3^, Joseph Boscarino^4^, Timothy Frankland^2^, Amy Loree^2^, Yong Hu^2^, Felicia Chi^1^

#### ^1^Kaiser Permanente Northern California Division of Research, Oakland, CA, USA; ^2^Kaiser Permanente Center for Health Research, Honolulu, HI, USA; ^3^Center for Health Policy & Health Services Research, Behavioral Health Services, Henry Ford Health System, Detroit, MI, USA; ^4^Geisiger, Canville, PA, USA

##### **Correspondence:** Stacy Sterling - stacy.a.sterling@kp.org

*Addiction Science & Clinical Practice* 2019, **14(Suppl 1):**A33

**Background:** Substance use (SU) problems significantly impact many adolescents. Early identification and intervention can be beneficial, yet clinical tools for facilitating identification are lacking. We developed and validated predictive models of adolescent SU problem emergence, using clinical and demographic data from four health systems.

**Methods:** This observational, electronic health records (EHR)-based retrospective cohort study identified a birth cohort of 41,176 adolescents, born between 1997 and 2000 in Kaiser Permanente Hawaii, KPHI; Kaiser Permanente Northern California, KPNC; Geisinger Health System, GHS and Henry Ford Health System, HFHS, with continuous membership since birth, allowing a 12-month gap. We examined data on demographics, socioeconomic status, diagnoses, risk behaviors, prescriptions and services utilization from 0 to 12, and data on diagnoses received by adolescents 12–18. The outcome was development of an SU use disorder between ages 12–18, defined as either: (1) a non-tobacco SU diagnosis, or (2) an SU treatment program contact. We used Cox regression models to develop a baseline model with child and maternal predictors occurring before age 12 as time-invariant predictors, and a series of time-varying models with final baseline model predictors plus diagnoses that teens received 12–18 as time-varying predictors.

**Results:** Age, gender, race/ethnicity and Medicaid-insured status, ADHD, conduct disorders, headache, oppositional defiant disorder and trauma/stress-related disorders before age 12 were associated with SU problems by 18, along with maternal SU disorders, major depression, and other depressive disorders. In the time-varying models, several early predictors were no longer significant if not also present between 12 and 18. Trauma/stress diagnoses, self-harm, injury/poisoning, and headache remained significant predictors even in the absence of later diagnoses.

**Conclusion:** Many early mental health problems alone are not predictive of adolescent SU disorders, but ongoing, persistent comorbidities seem to predict the development of adolescent AOD problems. Predictive models of this kind may inform targeted screening and intervention efforts.

## A34 Brief intervention for cannabis, alcohol, and sex-risk behaviors for adolescents in school-based health centers: comparison with assessment-only historical control

### Jan Gryczynski^1^, Shannon G. Mitchell^1^, Robert P. Schwartz^1^, Kristi Dusek^1^, Kevin E O’Grady^2^, Courtney D. Nordeck^1^

#### ^1^Friends Research Institute, Baltimore, MD, USA; ^2^University of Maryland, College Park, MD, USA

##### **Correspondence:** Jan Gryczynski - jgryczynski@friendsresearch.org

*Addiction Science & Clinical Practice* 2019, **14(Suppl 1):**A34

**Background:** School-based health centers (SBHCs) have emerged as important clinical settings in the US for expanding access to healthcare for underserved adolescents. SBHCs could hold promise as sites in which to deliver brief intervention (BI) for substance use.

**Methods:** Participants were adolescents aged 14–18 who screened positive for risky cannabis and/or alcohol use on the CRAFFT screener at two urban school-based health centers (SBHCs). A sample of adolescents were enrolled in a randomized trial of computer- vs. nurse practitioner-delivered BI (N = 300). Additionally, in the year prior to launching the trial, we enrolled an assessment-only cohort of adolescents using the same recruitment protocol and inclusion/exclusion criteria (N = 50). Participants completed assessments at baseline, 3-, and 6-month follow-up. The current study compared outcomes for the BI conditions with the historical assessment-only cohort. Frequency of cannabis, alcohol, unprotected sex, and sex while intoxicated at follow-up were examined using negative binomial regression, controlling for participant sex, age, clinic site, and baseline value of the outcome.

**Results:** There were no significant differences between computer- and nurse practitioner-delivered BI conditions on reported past-30-day frequency of cannabis, alcohol, unprotected sex, or sex while intoxicated. At 3-month follow-up, the pooled BI conditions had lower past-30-day frequency of alcohol use (IRR = .43; 95% CI = .29, .64; p < .001) and cannabis use (IRR = .74; 95% CI = .57, .97; p = .03) than the assessment-only cohort. At 6-month follow-up, the pooled BI conditions had lower frequency of alcohol use (IRR = .58; 95% CI = .34, .98; p = .04) and sex while intoxicated (IRR = .42, 95% CI = .21, .83; p = .01) than the assessment-only cohort.

**Conclusions:** Although we found no differences between two approaches to delivering BI at SBHCs on the outcomes considered, on average, participants who received a BI reported greater behavioral risk reductions than participants in a recent historical cohort that received no intervention.

**Trial registration:** NCT02387489.

## A35 A randomized trial of adolescent SBIRT in rural U.S. health centers: establishing 90 day substance use change in standard care participants

### Shannon G. Mitchell, Laura B. Monico, Jan Gryczynski, Robert P. Schwartz

#### Friends Research Institute, Baltimore, MD, USA

##### **Correspondence:** Shannon G. Mitchell - smitchell@friendsresearch.org

*Addiction Science & Clinical Practice* 2019, **14(Suppl 1):**A35

**Background:** The proposed presentation will include an overview of an on-going stepped-wedge randomized trial of adolescent SBIRT being conducted at 4 rural health centers in the United States. The study examines the effectiveness of delivering the full range of provider interventions using the FaCES (Facilitating Change for Excellence in SBIRT) approach. FaCES includes a prescribed set of responses (anticipatory guidance, abbreviated BI, full BI) based on S2BI screening results. Adolescent patients, age 12–17 years, will receive either the FaCES intervention or standard care, depending on when their provider is randomized to begin delivering the intervention.

**Methods:** As of March 31, 2019 a total of 621 patients had been recruited into the study across 4 rural U.S. health centers. Of those, 381 standard care control condition participants had also completed their 3-month follow-up interview, which included a re-administration of the S2BI assessing past 90 day substance use. Paired-samples t-tests were conducted to compare baseline with follow-up self-reported past 90 day use of tobacco, marijuana, and alcohol.

**Results:** Among the 381 standard care participants in the follow-up sample, 52% were female, 67% were white, and 34% were Hispanic. Significantly higher rates of alcohol use in the past 90 days were reported at follow-up (M = .366, SD = .66) than at baseline (M = .276, SD = .54); t(379) = − 3.4845, p = .0006. No significant differences were noted in past 90 day use rates of either tobacco or marijuana.

**Conclusions:** In order to interpret the effectiveness of adolescent SBIRT interventions it is important to include a standard care arm to track changes in substance use, which can fluctuate rapidly during adolescence. Early data indicate that reported alcohol use may increase for patients receiving standard care over the 3-month follow-up period.

## A36 Polysubstance use patterns and HIV disease severity among those with substance use disorder: Latent Class Analysis

### Nicolas Bertholet^1^, Michael Winter^2^, Timothy Heeren^3^, Alexander Walley^4^, Richard Saitz^5,6^

#### ^1^Addiction Medicine, Department of Psychiatry, Lausanne University Hospital and University of Lausanne, Lausanne, Switzerland; ^2^Biostatistics and Epidemiology Data Analytics Center, Boston University School of Public Health, Boston, Massachusetts, USA; ^3^Department of Biostatistics, Boston University School of Public Health, Boston, Massachusetts, USA; ^4^Clinical Addiction Research and Education Unit, Section of General Internal Medicine, School of Medicine and Boston Medical Center, Boston University, Boston, Massachusetts, USA; ^5^Clinical Addiction Research and Education Unit, Section of General Internal Medicine, School of Medicine and Boston Medical Center, Boston University, Boston, Massachusetts, USA; ^6^Department of Community Health Sciences, Boston University School of Public Health, Boston, Massachusetts, USA

##### **Correspondence:** Nicolas Bertholet - Nicolas.Bertholet@chuv.ch

*Addiction Science & Clinical Practice* 2019, **14(Suppl 1):**A36

**Background:** Polysubstance use is common among people living with HIV infection (PLWH) and substance use disorder (SUD) but its effects are under-studied. We aimed to (1) identify polysubstance use patterns over time with latent class analysis, and (2) assess their associations with HIV disease severity.

**Methods:** We studied a prospective cohort of 233 PLWH who also had SUD. Latent class analysis identified polysubstance use patterns based on the Alcohol Use Disorders Identification Test (consumption) and past 30-day use of cannabis, cocaine, opioids, and tranquilizers. We categorized changes in substance use patterns over 12 months and tested associations between those changes and CD4 cell count and HIV viral suppression at 12 months in linear and logistic regressions, adjusting for demographics.

**Results:** At baseline, three patterns (classes) were identified: 18% did not use any substance (NONE), 63% used mostly cannabis and alcohol (CA), and 19% used mostly opioids, cocaine, tranquilizers, cannabis and alcohol (MULTI). At 12 months, 61% were in the same class. Forty percent decreased the number of substances used (MULTI to CA, either to NONE) or remained as NONE; 43% were in CA both times; and 17% increased (NONE to CA or either to MULTI, including remaining MULTI). Adjusted mean CD4 count was lower among participants increasing substance use (mean [95% CI] 446 [318–574]) and among those in CA both times (464 [373–556]) compared to those who decreased or abstained throughout (605 [510–700], p = 0.005). No significant difference was observed for HIV viral suppression.

**Conclusions:** We identified distinct substance use patterns among PLWH and SUD: cannabis/alcohol, and opioids with alcohol and other drugs. Patterns changed over time, and changes towards fewer substances or no use were associated with better HIV disease severity (based on CD4 count). Findings may inform clinical advice for PLWH and SUD.

**Supported by:** U01AA020784/U24AA020778/U24AA020779/UL1TR001430.

## A37 Improved pain control and interpersonal relationships among older adults receiving buprenorphine therapy: results of a pilot study

### J. Paul Seale^1^, Amanda Abraham^2^, Samantha Harris^2^, J. Aaron Johnson^3^, Keerthika Ravikumar^1^, Omar Ahmad^1^, Mansi Amin^1^, Jorge del Rio^1^, Parth K. Patel^1^, Huma Rahman^1^, Kirk Von Sternberg^4^, Mary M. Velasquez^4^

#### ^1^Department of Family Medicine, Navicent Health, Macon, Georgia, USA; ^2^School of International and Public Affairs, University of Georgia, Athens, Georgia, USA; ^3^Institute of Public and Preventive Health, Augusta University, Augusta, Georgia, USA; ^4^Steve Hicks School of Social Work, University of Texas, Austin, Texas, USA

##### **Correspondence:** J. Paul Seale - seale.paul@navicenthealth.org

*Addiction Science & Clinical Practice* 2019, **14(Suppl 1):**A37

**Background:** While the U.S. opioid use disorder (OUD) epidemic affects large numbers of young adults, a significant minority of patients are older adults. The highest overdose rate is among those age 45–54. OUD in this population is poorly described. Recognition of barriers and bridges to treatment in older adults could increase treatment referrals.

**Materials and methods:** Structured interviews were conducted with 25 individuals age 45 and older receiving outpatient buprenorphine therapy in a small southern U.S. city.

**Results:** Mean age was 56 years. Mean age at first opioid use was 30.5. Most widely used opioids included hydrocodone (n = 23), oxycodone (23), methadone (4) and heroin (4). All met DSM 5 criteria for OUD (mean number of criteria 8.68). Six (24%) reported previous overdose. Most reported prior medication assisted treatment (MAT) with methadone (n = 12, 48%) or buprenorphine (n = 19, 76%). Referrals by family/friends were more common than physician/medical referrals [13 (52%) vs. 5 (20%)]. Patients’ concerns before starting MAT included how to control pain (58%), cost (50%), medication side effects (50%), and feeling MAT was “just another addiction” (42%). Reported benefits on MAT were regaining control of their lives (100%), absence of withdrawal (92%), improved relationships with friends/family (92%), better health (88%), decreased risk of overdose (88%), and less pain (80%). Most patients with chronic pain (20/21, 95%) reported pain control was equal or better than with other opioids.

**Conclusions:** Older adults with OUD are at significant risk of overdose. Concerns include pain control, cost, and potential side effects. In this pilot study, buprenorphine effectively managed pain for almost all patients. Clinicians providing SBIRT services should provide information regarding buprenorphine’s effectiveness in controlling chronic pain and common side effects. Policy makers should be urged to support access to MAT at low cost in order to increase the number of older adults accessing MAT.

## A38 Other drug use is common in hospital patients willing to start alcohol use disorder medication treatment: screening data from a comparative effectiveness trial

### Esperanza Romero-Rodriguez^1^, Susie Kim^1^, Clara Chen^1^, Debbie Cheng^1^, Henri Lee^2^, Tibor Palfai^3^, Jeffrey Samet^2^, Richard Saitz^1^

#### ^1^Boston University School of Public Health, Boston, MA, USA; ^2^Boston University School of Medicine, Boston, MA, USA; ^3^Boston University College of Arts and Sciences, Boston, MA, USA

##### **Correspondence:** Richard Saitz - rsaitz@bu.edu

*Addiction Science & Clinical Practice* 2019, **14(Suppl 1):**A38

**Background:** Drug use is common in people with alcohol use disorder (AUD) and opioid use can preclude use of naltrexone to treat it. The aim of this study was to describe substance use, and specifically opioid use, in adults with AUD, who were eligible to start naltrexone during hospitalization.

**Methods:** Adult inpatients with AUD (DSM5) and at least one past-month heavy drinking day (HDD) who had no naltrexone contraindications were enrolled in the Alcohol Disorder hOsPital Treatment (ADOPT) randomized trial comparing oral and extended-release naltrexone at discharge. AUD was assessed by the following: AUD and Associated Disabilities Interview Schedule-5 (AUDADIS-5); past 30-day alcohol use by the Timeline Followback; and past 3-month other drug use by the Alcohol, Smoking and Substance Involvement Screening Test (ASSIST).

**Results:** Of 821 patients screened who met AUD criteria, 11% were excluded due to opioid use. Among the first 176 participants enrolled, 82% were men, 48% black, 42% white; 13% were Hispanic; mean age was 50 ± 10 years. Participants reported mean 11 ± 11 standard (14 g) drinks/day, 20 ± 10 HDDs/month, mean percent HDD 68 ± 32. Almost half (48%) reported cannabis use and 27% reported cocaine use; 41% and 31%, respectively, had a moderate or high risk ASSIST specific substance involvement score.

**Conclusion:** Illicit drug use is common among medically hospitalized patients with alcohol use disorder. However, opioid use specifically only excludes a small minority of potential patients from receiving naltrexone for their AUD. Nevertheless, as drug use and disorder may affect prognosis and treatment selection, it should be considered in treatment planning.

## A39 Behavioral economics indices predict alcohol use and consequences in young men at 4-year follow-up – a target for brief intervention?

### Jacques Gaume^1^, James G. Murphy^2^, Gerhard Gmel^1^, Joseph Studer^1^, Jean-Bernard Daeppen^1^, Nicolas Bertholet^1^

#### ^1^Lausanne University Hospital, Lausanne, Switzerland; ^2^University of Memphis, Memphis, TE, USA

##### **Correspondence:** Jacques Gaume - jacques.gaume@chuv.ch

*Addiction Science & Clinical Practice* 2019, **14(Suppl 1):**A39

**Background:** According to the behavioral economics framework, substance use is more likely when constraints on use are minimal and when there are important constraints on access to substance-free reinforcers. For example, alcohol is a potent reinforcer, but its consumption is sensitive to constraints on access (including drink price) and the presence of alternative reinforcers. The alcohol purchase task (APT) presents a scenario and asks participants how many drinks they would purchase and consume at different prices. It has been used among students and small clinical samples and has not been tested using long-term prospective design.

**Methods:** We administered the APT to a large sample of 4790 Swiss young men from the general population. Among those, 4326 (90.3%) were successfully followed-up 4 years later [mean age 21.4 and 25.4 (sd = 1.3)]. Parameters derived from the APT at baseline were used to predict weekly drinking, monthly binge drinking, maximum drinks in one occasion, alcohol-related consequences, and DSM-5 alcohol use disorder criteria.

**Results:** Intensity (planned consumption when drinks are free) and Omax (maximum alcohol expenditure) were significantly correlated with all outcomes (r range: 0.25–0.37 for Intensity, 0.17–0.28 for Omax, all p < 0.001). Breakpoint (price at which consumption was suppressed) and Elasticity were significantly, but weakly correlated with outcomes (r range: 0.09–0.12 for Breakpoint, − 0.07 to 0.11 for Elasticity, all p < 0.001). Pmax (price at which demand became elastic) was not a significant predictor. Regression analyses controlling for baseline value of outcome showed consistent findings.

**Conclusions:** Behavioral economics measures are useful in characterizing alcohol demand in young men from the general population and have long-term predictive value. Integrating behavioral economics components in BI models has been proposed but seldom tested. Potential for this approach will be discussed.

## A40 Analyzing profiles of co-occurring risk among low-income SBIRT patients engaged in federally-qualified health centers in the United States

### Michael A. Lawson^1^, Shanna A McIntosh^2^, Lauren Holmes^2^, David L. Albright^2^, Jacqueline Doss^2^

#### ^1^College of Education, University of Alabama, Tuscaloosa, AL, USA; ^2^School of Social Work, University of Alabama, Tuscaloosa, AL, USA

##### **Correspondence:** Michael A. Lawson - malawson1@ua.edu

*Addiction Science & Clinical Practice* 2019, **14(Suppl 1):**A40

**Background:** The aim of this study is to analyze how substance use and mental health risks present themselves among different sub-populations of patients who “screen in” for SBIRT services. A secondary purpose is to explore how analyses of patient risk can be used by SBIRT program staff to enhance clinical intervention and training. The setting for the study is an SBIRT program serving low-income, Federally Qualified Health Centers (FQHC) in the Southeastern United States.

**Methods:** Latent Class Analysis (LCA) was used to model different profiles of substance use and mental health risk/difficulty among 600 patients with qualifying DAST-10 or Audit scores. These models were estimated using the DAST-10, the Audit, and the PHQ-9 for depression. In addition, patient age, race/ethnic status, and sex (as a binary) were analyzed for their association with each risk profile.

**Results:** Median age = 42 (range 20 to 72), 42% women 38% white, 62% African American. LCA yielded three characteristically different risk profiles among patients with qualifying AUDIT/DAST-10 scores. These profiles included a “Drugs Only” class (70%) comprised of patients who screened-in for brief intervention. A second risk profile, the “Dual Vulnerability” (25%) class, included patients who engaged in regular binge drinking and had DAST-10 scores that qualified them for brief intervention. The third risk profile, the “Severe Vulnerability” class (5%), was indicated by severe alcohol abuse, related behavioral difficulty, depression, and qualifying DAST-10 scores. Last, our regression analyses indicated that these profiles could be partially differentiated by patient age, sex, and ethnicity.

**Conclusions:** These sub-population profiles, together with their respective correlates, can be used by program staff to improve clinical practice and training. Specifically, an analysis of interview data suggested that these profiles were useful in helping program leaders develop modified scripts for motivational interviewing and client engagement among qualifying patients.

## A41 Screening and brief interventions to reduced unhealthy alcohol use and improved subjective mental health: results of Prague RCT study

### Ladislav Czemy, Bohumil Seifert, Zuzana Dvorakova

#### Centre of Epidemiological and Clinical Research in Addictions, National Institute of Mental Health, Klecany, Czech Republic

##### **Correspondence:** Ladislav Czemy - csemy@nudz.cz

*Addiction Science & Clinical Practice* 2019, **14(Suppl 1):**A41

**Goal:** To evaluate the effects of brief intervention to reduce unhealthy alcohol use in primary care setting.

**Background:** The Czech Republic belongs to countries with high per capita alcohol consumption. Screening and brief intervention in primary health care may reduce harmful alcohol consumption.

**Materials and methods:** 161 patients (61% were males) of 699 were identified as hazardous or harmful drinkers in ten offices of general practitioners using the ASSIST v.3 or AUDIT‐C screening tests. Patients were assigned into intervention group 1 (intervention provided by the doctor) and intervention group 2 (only feedback from screening and self‐help materials were provided).The baseline mean alcohol consumption calculated BSQF method was compared with consumption after 3 months.

**Results:** The results showed that in the intervention group 1 there was a statistically significant decrease in the total mean alcohol consumption from 262.6 grams of alcohol per week to 175.2 g (t = 3.64; df = 89; p < .001; Cohen‘s d = 0.36). With the exception of consumption of spirits, a statistically significant decrease in consumption was found in beer (from 162.5 to 101.5 grams per week) and wine (from 92.5 to 52.5 grams per week). Total alcohol consumption also decreased in the intervention group 2 from 194.6 to 155.0 grams per week, but the difference in means did not reach statistical significance (t = 1.45; df = 32; p = 0.158). Subjectively perceived mental health improved in group 1 as well.

**Conclusions:** The results suggest that brief interventions implemented in primary care are effective and can be recommended for implementation into primary health care.

## A42 An application of deviance regulation theory to increase safe drinking strategies in first year students

### Robert Dvorak, Angelina Leary

#### Department of Psychology, University of Central Florida, Orlando, FL, USA

##### **Correspondence:** Robert Dvorak - robert.dvorak@ucf.edu

*Addiction Science & Clinical Practice* 2019, **14(Suppl 1):**A42

**Background:** Many students come to university relatively inexperienced with alcohol, which may increase alcohol-related consequences. Several interventions exist to combat this problem. However, recent research suggests these interventions may not be as successful as initially thought. The current study investigates the use of a relatively new web-based intervention, grounded in Deviance Regulation Theory (DRT), aimed at increasing alcohol Protective Behavioral Strategies (PBS).

**Methods:** College freshmen participants (n = 157) were randomly assigned to one of three conditions: a positive message about individuals who use PBS, a negative message about individuals who do not use PBS, or an attention control condition. Participants then completed weekly assessments examining alcohol-related behaviors for six weeks. Participants also reported norms of PBS use each week.

**Results:** Findings replicated previous research, showing actual PBS use increases across time among those with initially high PBS norms who also received a negative message about non-PBS use (b = 0.120, p = .022). Further, there was an increase in PBS norms across time (b = 0.023, p = .019). The growth in the effectiveness of the negative message was related to increases in PBS norms across time (r = .129, p < .001). There were no immediate effect of the positive message. However, within-subjects analyses showed that within a given week, the positive message was associated with increased weekly PBS among those with low weekly PBS norms, a finding consistent with DRT prediction.

**Conclusion:** These results suggest that DRT works by (a) increasing PBS use across time among those who receive a negative message by also increasing PBS norms and (b) increasing PBS use at the event level as a function of current PBS normative beliefs. The results indicate an in-the-moment DRT intervention may be beneficial for first-time-in-college students.

## A43 Pharmacy undergraduates’ alcohol use and perceptions to supporting those with alcohol problems

### Aliya Khan, Ranjita Dhital

#### Pharmacy Department, University of Reading, Reading, UK

##### **Correspondence:** Ranjita Dhital - r.dhital@reading.ac.uk

*Addiction Science & Clinical Practice* 2019, **14(Suppl 1):**A43

**Background:** Young people (18–24 years) are more likely to ‘binge drink’ and increase their health risks than other age groups. There is growing evidence that health staffs’ attitudes affect alcohol service delivery. This research aims to identify alcohol use amongst UK final-year pharmacy undergraduates and explore if alcohol consumption and other characteristics are related to perceptions to supporting those with alcohol problems.

**Methods:** Mixed-methods approach was used to: screen students’ drinking using the Alcohol Use Disorders Identification Test (AUDIT); measure attitudes to support patients using a modified version of the Short Alcohol and Alcohol Problems Perception Questionnaire (SAAPPQ); and explore students’ perceptions toward supporting drinkers through a focus group.

**Results:** 54 students (44 female, age-range 21–25 years) from 106 participated (51% response). Mean AUDIT was 3.46 [S.D. ± 4.58]; 19% (N = 10) were higher risk (AUDIT ≥ 8), mean AUDIT = 11.4 [S.D. ± 3.01]; and 82% (N = 44) were low risk drinkers (AUDIT ≤ 7), mean AUDIT = 1.66 [S.D. ± 2.36]. Median total attitudes (most positive = 7 and least positive = 1) was 3.8 (range 4.2 to 3.5), close to neutral. Non-parametric tests identified higher-risk drinkers had significantly higher work-satisfaction (P = 0.013) and total positive attitudes toward this patient group (P = 0.013) compared to low-risk drinkers. Also, smokers (N = 8) had significantly higher work-satisfaction (P = 0.034) and total positive attitudes (P = 0.034) compared to non-smokers (N = 46). Focus group (involving 8 students) identified themes relating to work environment, social influences, alcohol education and stigma as possible factors relating to alcohol use and support.

**Conclusions:** Most students were low-risk drinkers with neutral attitudes to supporting patients. Students wanted further alcohol education and counselling skills during their pharmacy degree to support this patient group for future clinical practice. Smokers and higher-risk drinkers had more positive attitudes. These findings require further examination, especially if experience of alcohol use and smoking may enhance knowledge and relatability to drinkers.

## A44 Comorbid depression in alcohol users: refining target groups for brief alcohol interventions in medical care settings

### Diana Guertler^1,2^, Anne Moehring^1,2^, Kristian Krause^1^, Jennis Freyer-Adam^2,3^, Sabina Ulbricht^1,2^, Gallus Bischof^4^, Hans-Jürgen Rumpf^4^, Anil Batra^5^, Sandra Eck^5^, Sophie Baumann^1,2,6^, Ulrich John^1,2^; Christian Meyer^1,2^

#### ^1^Institute of Social Medicine and Prevention, University Medicine Greifswald, Greifswald, Germany; ^2^DZHK (German Center for Cardiovascular Research), Partner Site Greifswald, Greifswald, Germany; ^3^Institute for Medical Psychology, University Medicine Greifswald, Greifswald, Germany; ^4^Department of Psychiatry and Psychotherapy, University of Lübeck, Lübeck, Germany; ^5^Department of Psychiatry and Psychotherapy, University Hospital of Tübingen, Tübingen, Germany; ^6^Institute and Policlinic for Occupational and Social Medicine, Technische Universität Dresden, Germany

##### **Correspondence:** Diana Guertler - guertlerd@uni-greifswald.de

*Addiction Science & Clinical Practice* 2019, **14(Suppl 1):**A44

**Background:** Medical care provides a suitable setting to reach large numbers of alcohol users. As comorbid depression adds to the maintenance of risky alcohol use, the effectiveness of brief interventions (BI) might be enhanced by adjusting them to depression severity. The aim of this paper was to estimate comorbid depressive symptom severity and presence of major depression along the full continuum of alcohol use.

**Methods:** Medical care patients aged 18–64 years were proactively approached for an anonymous health screening (participation rate = 87%, n = 12,828). Continuous alcohol use measures were derived from an expanded Alcohol Use Disorder Identification Test (AUDIT): alcohol consumption per day and occasion, excessive consumption days and the AUDIT sum score. Depressive symptoms for the worst 2-week period in the last year were assessed with the Patient Health Questionnaire (PHQ-8). Negative binomial and logistic regression analyses were used to predict depressive symptom severity (PHQ-8 sum score) and presence of major depression (PHQ-8 sum score ≥ 10). Fractional polynomials were applied to model potential non-linearity.

**Results:** Data revealed curvilinear relationships of depressive symptom severity and presence of major depression with all alcohol use measures after controlling for socio-demographics and health behaviors (*P *< 0.05). Lowest depressive symptom severity and odds of major depression were found for alcohol consumptions of 1.1 g per day, 10.5 g per occasion, one day of excessive consumption per month, and those with an AUDIT sum score of 2. Higher values were found for abstinence and higher consumption levels. Women and younger individuals showed higher depression outcomes along the full continuum of alcohol use with steeper risk slopes compared to men and older individuals.

**Conclusions:** Increases of depressive symptom severity and odds of major depression were already evident in alcohol users drinking below the established limits for low risk drinking. Findings demonstrate the public health relevance of screening for depressive symptoms within alcohol users. Especially women and young individuals may benefit from dual focus BI.

## A45 Hazardous alcohol consumption in depressed health care patients – associations with health-related quality of life

### Kristian Krause^1^, Diana Guertler^1,2^, Anne Moehring^1,2^, Gallus Bischof^3^, Hans-Jürgen Rumpf^3^, Anil Batra^4^, Thomas Kohlmann^5^, Ulrich John^1,2^, Christian Meyer^1,2^

#### ^1^Institute of Social Medicine and Prevention, University Medicine Greifswald, Greifswald, Germany; ^2^DZHK (German Center for Cardiovascular Research), Partner Site Greifswald, Greifswald, Germany; ^3^Department of Psychiatry and Psychotherapy, University of Lübeck, Lübeck, Germany; ^4^Department of Psychiatry and Psychotherapy, University Hospital of Tübingen, Tübingen, Germany; ^5^Institute for Community Medicine, University Medicine Greifswald, Greifswald, Germany

##### **Correspondence:** Kristian Krause - kristian.krause@uni-greifswald.de

*Addiction Science & Clinical Practice* 2019, **14(Suppl 1):**A45

**Background:** Previous research revealed that individuals with alcohol use problems and comorbid depression or anxiety report less health-related quality of life (HRQOL) than those with alcohol use problems alone. Here we analyze how the presence of comorbid hazardous alcohol consumption is associated with HRQOL in depressed health care patients (HCPs).

**Method:** Consecutive HCPs from ambulatory practices and general hospitals were systematically screened. We recruited 589 participants (61.5% female, mean age 39.3 years) reporting an episode with clinical or subclinical depressive symptoms in the past year. Among those, 134 (22.8%) reported hazardous alcohol consumption. HRQOL was measured by the Veterans Rand 12 Item Health Survey (VR-12). Multivariate regression analyses controlling for sociodemographics and recruitment setting were conducted to analyze associations between hazardous alcohol consumption and the physical (PCS) and mental component score (MCS) of the VR-12.

**Results:** Hazardous alcohol consumption was significantly associated with the PCS (*Beta *= .118, *p *= .001) but not with the MCS (*Beta *= .048, *p *= .255) of the VR-12 when controlling for covariates. Regarding the PCS subscales, significant associations were found for *general health perception* (*Beta *= .126, *p *= .001), *physical functioning* (*Beta *= .099, *p *= .008), and *bodily pain* (*Beta *= .105, *p *= .008). Among the MCS subscales, significant associations were found for *energy/vitality* (*Beta *= .134, *p *= .002) and *social functioning* (*Beta *= 1.28, *p *= .002).

**Discussion:** Hazardous alcohol consumption was associated with better HRQOL in depressed HCPs. This could imply that hazardous consumption was used to effectively cope with depressive symptoms. However, the results should be interpreted with caution. Due to the cross-sectional nature of our data, causal interpretations are precluded. Furthermore, non-linear associations between depression and alcohol consumption may have existed. Our analysis, however, was restricted to linear associations.

## A46 Computerized screening and clinical decision support can increase primary care provider delivery of brief intervention for unhealthy drug use: baseline results from a pilot study of the Substance Use Screening and Intervention Tool (SUSIT)

### Jennifer McNeely, Medha Mazumdar, Antonia Polyn, Steven Floyd, Akarsh Sharma, Donna Shelley, Charles Cleland

#### Dept. of Population Health, NYU School of Medicine, New York, NY USA

##### **Correspondence:** Jennifer McNeely - jennifer.mcneely@nyulangone.org

*Addiction Science & Clinical Practice* 2019, **14(Suppl 1):**A46

**Background:** Primary care providers (PCPs) face multiple barriers to offering substance use interventions, including lack of time, knowledge, and information about their patients’ drug use. We developed a tablet-based Substance Use Screening and Intervention Tool (SUSIT) to assist PCPs by delivering screening results and clinical decision support for conducting brief intervention (BI) to address unhealthy drug use. The SUSIT screener is a self-administered brief screen (SUBS) and modified WHO-ASSIST. This pilot study examined whether the SUSIT increases delivery of BI during primary care visits.

**Methods:** Adult patients completed tablet-based screening in the waiting room, and identified their drug of most concern (DOMC). Those with moderate-risk use of any drug (without high-risk alcohol or drug use) were eligible. A pre-post design compared participants enrolled during the control period to a new group of participants enrolled during the intervention period, in which PCPs received the SUSIT. All participants completed an after-visit survey documenting the elements of BI delivered by the PCP, and a 90-day timeline follow-back.

**Results:** The 78 participants (42 control, 36 intervention) were majority male (76%), with a mean age of 46 (SD = 13). Marijuana was the most prevalent DOMC (n = 52 (66.7%)); cocaine was the second most prevalent DOMC (n = 7 (9.0%)). Mean days of use of the DOMC in the past 90 days was 38.8 (SD = 37.7). During the intervention period, PCPs used the SUSIT with 31 of 36 (86%) participants. Participants in the intervention condition were more likely to report receiving BI [(n = 33 (91.7%) vs. n = 17 (40.5%), P < 0.001]. The intervention group also received more elements of BI [median = 9.5, mean 7.8 (SD = 4.5) vs. median = 0, mean 2.7 (SD = 4.3); P < 0.001].

**Conclusions:** Providing drug use screening information and clinical decision support to PCPs increased the delivery of BI during routine primary care visits. Future analyses will examine changes in drug use behavior 3 months post-intervention.

**Trial registration:** ClinicalTrials.gov registration number: 16-01074.

## A47 Consumption of psychoactive substances in tuberculosis patients: interface to adherence to treatment and brief intervention

### Sônia Suelí S. E. Santo^1^, Angela Maria M. Abreu^1^, Luciana F. Portela^2^, Larissa R. Mattos^1^

#### ^1^Department of Public Health, Anna Nery School of Nursing, Federal University of Rio de Janeiro, Rio de Janeiro, RJ, Brazil; ^2^Evandro Chagas Infectology Institute, FIOCRUZ, Rio de Janeiro, RJ, Brazil

##### **Correspondence:** Sônia Suelí S.E. Santo - sucessonia@gmail.com

*Addiction Science & Clinical Practice* 2019, **14(Suppl 1):**A47

**Background:** Association of the use of psychoactive substances and tuberculosis make diagnosis and treatment a barrier to reach universal coverage of the disease worldwide.

**Objectives:** To identify the profile and the pattern of consumption of psychoactive substances of patients undergoing tuberculosis treatment in the network of basic health services; to analyse the adherence to the treatment of patients of tuberculosis who consume these substances and perform brief intervention in this clientele from the perspective of the adherence to the tuberculosis treatment.

**Methods:** Sectional study, carried out in primary care units in the modality of the Family Health Strategy, in Rio de Janeiro, with a sample of 114 patients in the treatment of tuberculosis using the ASSIST. The exposure variable was the consumption of psychoactive substances and the outcome variable adherence to treatment. In the first phase the brief intervention was carried out, in the stages of feedback, due guidance and empathically. In the second phase after two months, a search was performed on the medical record for confirmation or non‐compliance.

**Results:** Prevalence in the male population 71.1%, median age 39 years, incomplete primary schooling 52.6%, brown skin color 42.1%, family income > 1 minimum wage, 74.5% lived with relatives. Prevalence for tobacco 28.0%, alcoholic beverages 12.3%, marijuana 5.4% and cocaine/Crack 3.5%. Regarding adherence, after two months of Brief Intervention, with a survey in the patients’ charts, a higher prevalence of adherence was observed in the male population, over 40 years old, with medium/high school education, married and living in union, whites received up to 1 minimum wage, live with relatives, adhered to the treatment of tuberculosis.

**Conclusions:** These results demonstrate the importance of brief interventions applied by health Professionals with these patients, decreasing the incidence of infected and bacilliferous patients, prone to the spread of the disease.

## A48 Prevalence of alcohol misuse problem (AMP) recognition within those meeting criteria for alcohol misuse: A meta-analysis

### Panagiotis Spanakis^1^, Jessica Smith^1^, Rachael Gribble^2^, Sharon Stevelink^2^, Roberto Rona^2^, Nicola Fear^2^, Laura Goodwin^1^

#### ^1^University of Liverpool, Liverpool, UK; ^2^King’s College London, London, UK

##### **Correspondence:** Panagiotis Spanakis - spanak87@liverpool.ac.uk

*Addiction Science & Clinical Practice* 2019, **14(Suppl 1):**A48

**Background:** Recognition of AMP is a fundamental stage in the psychological process of taking action to change behaviour and, if necessary, seek treatment. Several studies have identified that AMP recognition is a significant correlate of help-seeking, while lack of recognition is a barrier to help. A systematic review and meta-analysis were conducted to estimate the prevalence of AMP recognition within individuals meeting validated AMP criteria.

**Methods:** We searched PsycInfo, Web of Science, Scopus and MedLine using the keywords: problem*; recogni* OR perceive* OR perception OR self-identif*; alcohol. We identified studies that reported weighted or unweighted frequencies of individuals who meet criteria for AMP (e.g. AUDIT scores or DSM criteria) and who self-identified their problems, self-recognised a need for receiving help, or have passed the pre-contemplation stage for taking action on their problems. Studies were eligible if they were published between 2000 and 2019 in English and included an adult sample. Studies were excluded if the study used a sample of adolescents, university students, or illicit drug users. A random-effects model meta-analysis was used to estimate the pooled prevalence of AMP recognition with 95% CIs.

**Results:** 27 papers were included in the meta-analysis (N = 77,081, on average 73% were male). Seventeen studies included participants with at least hazardous drinking and ten studies included participants with alcohol use disorder (AUD). Thirteen studies examined AMP recognition directly (self-identification), eight studies examined stage of change, and six studies examined need for help. Preliminary results showed that the pooled prevalence of AMP recognition was 42% (95 CI = 34%–51%).

**Conclusion:** Less than half of those with AMP recognise their problems. Given the importance of AMP recognition in the process of change, future research should focus on policies and interventions that could help the affected individuals increase their self-awareness regarding their AMP.

## A49 Designing an alcohol brief intervention targeted at the unemployed

### Mike Jecks^1^, Laura Goodwin^1^, Mark Gabbay^1^, Caryl Beynon^2^, Matt Field^3^

#### ^1^University of Liverpool, Liverpool, UK; ^2^Public Health England, Liverpool, UK; ^3^University of Sheffield, Sheffield, UK

##### **Correspondence:** Mike Jecks - mjecks@liverpool.ac.uk

*Addiction Science & Clinical Practice* 2019, **14(Suppl 1):**A49

**Background:** Problematic drinking is associated with unemployment, and this association may be bidirectional (Department for Work and Pensions 2015). Unemployment has been associated with depression (Zuelke et al. 2018) as well as boredom (De Witte et al. 2012) which in turn may be linked to increased alcohol consumption (Mercer-Lynn et al. 2011; Patrick and Schulenberg 2011) as well as increased likelihood of relapse in alcohol and substance use (Corvinelli 2005). This paper aims to investigate the potential links between alcohol use, unemployment, and drinking motives including boredom, with a view to designing an Alcohol Brief Intervention for those out of work based on this information.

**Methods:** The study recruited employed (n = 94) and unemployed (actively seeking employment) (n = 94) individuals through social media. The groups were compared on their drinking habits (Alcohol Use Disorder Identification Test), drinking motivations (Drinking Motivations Questionnaire), and recent feelings of boredom and low mood. MANOVAs were run to explore the between group differences, with adjusted regressions examining the role of potential confounders.

**Results:** The average age was 33.05 (SD = 10.902) years, with more females (73.5%) recruited. Unemployed participants scored significantly higher on the AUDIT [F(1,164) = 8.59, p < 0.01], coping [F(1,164) = 9.80, p < 0.01], and boredom[F(1,164) = 14.83, p < 0.01] drinking motivations compared to the employed group. These motivations were significantly positively associated with higher AUDIT scores in the unemployed group when controlling for demographic, depression, and boredom scores.

**Conclusion:** The study shows that problematic alcohol use is more common in the unemployed and demonstrates the links between alcohol use and unemployment, with boredom and coping as two key motives among the unemployed for increased drinking. Further qualitative work will seek to engage those who are out of work to further understand the link between boredom and alcohol use in the unemployed. This will then be used to develop an Alcohol Brief Intervention which will be targeted at the unemployed.

## A50 Adaptation, for Indigenous teenagers, of an alcohol and drugs screening test: the DEP-ADO

### Jennifer Beauregard^1^, Myriam Laventure^1^, Chantal Plourde^2^, Joël Tremblay^2^

#### ^1^Université de Sherbrooke, Sherbrooke, Canada; ^2^Université du Québec à Trois-Rivières, Trois-Rivières, Canada

##### **Correspondence:** Jennifer Beauregard - jennifer.beauregard@usherbrooke.ca

*Addiction Science & Clinical Practice* 2019, **14(Suppl 1):**A50

**Background:** In order to improve interventions efficiency among Indigenous teenagers, adapting questionnaires to their cultural reality is paramount. In fact, without this cultural adaptation, the results obtained with these questionnaires could minimize or exaggerate the extent of youth’s difficulties. The present study adapted the Detection of Alcohol and Drug Problems among Teenagers (Landry et al. 2004).

**Method:** Crees (anglophones) and Attikamekws (francophones) have collaborated to the validation of this screening test. A first step constituted in a co-building process allowing for the development of an initial version of the DEP-ADO adapted to Indigenous’ cultural reality. During a second step, 20 youth have filled the adapted version and have taken part in focus groups aimed to give them the opportunity to comment their understanding and usefulness of such a grid for Indigenous Youth. Finally, during a third step, a second adapted version of the test was administered to Youth from three communities (N = 421, average age 14.75) for a final validation.

**Results and conclusion:** The main challenge was to make sure that the wording and the examples were easily understandable and make sense for Indigenous Youth. For examples: changing the questionnaire response options, give examples to help understand the issues at stake, review the phrasing of the questions. During the focus groups, youth reported that many questions or concepts were difficult to understand and needed to be reworded or clarified. Also, as some questions are more sensitive, participants reported experiencing shame and hesitating to answer accurately. Regarding the reliability, the DEP-ADO (adapted) scales indicated that alcohol scale (α tet = 0.91), drugs scale (α tet = 0.86), risk factors scale (α tet = 0.88) and global score (α tet = 0.93) had adequate reliability for Indigenous Youth. The one-factor latent structure was confirmed for all scales.

## A51 Implementation of a Parenthood and Addiction Program: programs based on probative data vs practices based on probative data

### Myriam Laventure, Marie-Josée Letarte, Jennifer Beauregard

#### ^1^Université de Sherbrooke, Sherbrooke, Canada

##### **Correspondence:** Myriam Laventure - Myriam.Laventure@USherbrooke.ca

*Addiction Science & Clinical Practice* 2019, **14(Suppl 1):**A51

**Background:** Less than 10% of the organizations treating addiction include parenthood support in their treatment plan. The Parent Management Training (PMT) programs have proven their efficiency among the parents who have an addiction. These programs based on probative data and relying on the best practices are, however, not easy to implement in some practice environments. Two approaches are in opposition; the one of a program type based on probative data and the one of a practice type based on probative data. How can we maintain the equilibrium between the «ingredients» associated to a program’s efficiency and the implementation in all the different practice environments?

**Method:** Developed in order to meet the specific needs of the families in which parents are addicted to alcohol or drugs, the PMT *Cap sur la famille* was implemented in addiction treatment centers in Quebec. However, in consideration of regional particularities, the program could not be implemented in its entirety uniformly. A consultation was carried out in order to identify the factors that could hamper the implementation of this type of program in the practice environments. In total, 17 addiction treatment centers (22 standardized interviews) have been consulted.

**Results and conclusion:** During consultation and despite the acknowledgement of the need for a PMT program specific to the clientele in addiction, managers and addiction workers agreed that the implementation feasibility of the PMT program presents important challenges on human resources and facilities aspects. Up to now, 8 addiction treatment centers are implementing the program, each with its adapted formula. Aspects such as dosage, intensity, content and clientele reached needed to be adapted while respecting the best practices recommendations. The steps taken, the various versions of the program and the implementation quality will be discussed according to the practice based on probative data.

## A52 Association of perceived parental disapproval with marijuana and alcohol use among urban US high school students receiving brief intervention

### Courtney D. Nordeck, Kristi Dusek, Shannon G. Mitchell, Robert Schwartz, Jan Gryczynski

#### Friends Research Institute, Baltimore, MD, USA

##### **Correspondence:** Courtney D. Nordeck - cnordeck@friendsresearch.org

*Addiction Science & Clinical Practice* 2019, **14(Suppl 1):**A52

**Background:** Adolescent substance use poses serious physical and mental health risks that can extend into adulthood. Parental disapproval of substance use is commonly considered an important protective factor for prevention of risky adolescent behaviors. Less is known about the role of parental disapproval among youth who have initiated risky substance use and are candidates for brief intervention.

**Methods:** Adolescents ages 14–18 with risky alcohol and/or marijuana use were recruited from two US urban school-based health centers into a randomized trial comparing a computer versus nurse practitioner-delivered brief intervention. This secondary analysis examines the relationship between perceived parental disapproval of substance use and adolescents’ frequency of marijuana and alcohol use at baseline, 3-, and 6-month follow-up. Measures of parental disapproval were aligned with questions from national epidemiological survey data. Generalized estimating equations were used to examine trajectories of alcohol and marijuana use frequency by level of perceived parental disapproval, adjusting for identified covariates.

**Results:** Compared to national norms, youth in this sample reported low parental disapproval of substance use. Nevertheless, perceived parental disapproval was associated with fewer days of marijuana use (ps < 0.01; e.g., at baseline, mean [SD]: 14.39 [11.39] versus 9.96 [9.73] days). The relationship between parental disapproval and frequency of alcohol use was non-significant (p = 0.06). Level of perceived parental disapproval did not yield different trajectories of use frequency over time for marijuana (p = 0.59) or alcohol (p = 0.92), nor did the brief interventions perform differently based on level of perceived parental disapproval (p = .90 and .29 for marijuana and alcohol, respectively).

**Conclusions:** Perceived parental disapproval of substance use may play a role in tempering frequency of marijuana use, even among youth who already meet risk thresholds for intervention. Degree of parental disapproval may be important to consider in the design of brief interventions for adolescents to reduce substance use and promote healthy adjustment.

## A53 Barriers to the implementation of brief interventions in primary care centers for addictions in Northwest Mexico

### Ana L. Jimenez-Perez^1^, Eunice Vargas-Contreras^1^, Kalina I. Martinez-Martinez^2^, Marcela Tiburcio-Sainz^3^

#### ^1^Universidad Autonoma de Baja California, Ensenada, Baja California, Mexico; ^2^Universidad Autonoma de Aguascalientes, Aguascalientes, Mexico; ^3^Instituto Nacional de Psiquiatria Ramon de la Fuente Muniz, Ciudad de Mexico, Mexico

##### **Correspondence:** Eunice Vargas-Contreras - eunice.vargas@uabc.edu.mx

*Addiction Science & Clinical Practice* 2019, **14(Suppl 1):**A53

**Background:** In Mexico, there are 324 Primary Care Centers for Addictions (CAPA) responsible for providing services to individuals with problematic alcohol or drug use. National practice guidelines recommends the delivery of Brief Interventions (BI) as a means to address substance use‐related problems. However, in the Northwest of Mexico, as in the rest of the country, BIs have not yet been routinely implemented.

**Methods:** The objective of this descriptive study was to determine which factors hinder the implementation of BI from the perspective of 102 therapists working at 17 different CAPA located in the Northwest of Mexico. A questionnaire with internal consistency level of alpha .89 was used to collect data.

**Results:** The results suggest that the frequency with which participants read scientific documents during their time as addiction therapists, as well as their knowledge about BI, explain 68.55% of the variance of their use of BI. However there is also limited information on the content of the treatment currently provided by therapists in this region. Although substance use treatment has been provided by CAPAs for over 12 years, there has not yet been any evaluation of existing provision by local authorities.

**Conclusions:** We conclude that it is necessary to develop relevant strategies to stimulate the use of BI in CAPAs, including face‐to‐face training, and feedback to demonstrate the benefits associated with implementation. It is also necessary to strengthen local health policies to better support providers to deliver BI for substance use, in order to reduce the high consumption of alcohol and other drugs in the region.

## A54 Experiences of encountering outpatients with problematic substance use in the psychiatric context

### Elisabeth Petersén, Anna Thurang, Anne H. Berman

#### Centre for Psychiatry Research, Department of Clinical Neuroscience, Karolinska Institutet, & Stockholm Health Care Services, Stockholm County Council, Stockholm, Sweden

##### **Correspondence:** Elisabeth Petersen - elisabeth.petersen@ki.se

*Addiction Science & Clinical Practice* 2019, **14(Suppl 1):**A54

**Background:** High co-morbidity is known to exist between mental illness and problematic substance use. In patients seeking psychiatric care, 20–30% have been found to have problematic substance use, most often alcohol. Living with problematic alcohol consumption can contribute to a sense of exclusion, a sense of not complying with society’s norms, where seeking help for problematic alcohol consumption can be experienced as stigmatizing and challenging for the patient. Offering effective digital interventions for reducing alcohol consumption among patients in psychiatry might help reduce the feeling of stigma or shame and lead to more patients seeking help.

**Methods:** Exploratory study with a qualitative design. Data from interviews with outpatient psychiatry clinic directors and focus groups with clinical staff were evaluated using content analysis inspired by phenomenological-hermeneutic methodology.

**Results:** The experience of encountering patients with problematic substance use and elucidating visions of digital interventions resulted in three themes with corresponding sub-themes: Bridging the organizational gap was illuminated by: having an established collaboration and facing difficulties in the collaboration. Having beliefs about the patient you encounter was illuminated by: working with stigmatized patients and stigmatizing the patient. Striving to achieve a therapeutic alliance was illuminated by: having a feeling of developing together and supporting the patient towards recovery.

**Conclusions:** Caring for patients with problematic substance use was perceived by psychiatric health care professionals as difficult. They felt that a lack of resources and knowledge were obstacles in their work. Cooperation between psychiatry and dependency care often led to problems. Being able to work with digital screening and digital interventions in the future was perceived by the participants as positive and as a potential opportunity to overcome the gap between psychiatry and dependency care.

## A55 Brief intervention to motivate behaviour change in women with risky and harmful alcohol use

### Divane de Vargas, Talita Dutra Ponce

#### Sao Paulo University, School of Nursing, Sao Paulo, Brazil

##### **Correspondence:** Divane de Vargas - vargas@usp.br

*Addiction Science & Clinical Practice* 2019, **14(Suppl 1):**A55

**Background:** A significant number of women attending Primary Health Care (PHC) services are drinking at risky or harmful levels, with increased prevalence of problematic consumption patterns in this population in recent years. Therefore, the delivery of coping strategies and prevention measures in PHC settings has been suggested, including Brief Interventions (BI) for alcohol. BI focuses on increasing patients’ motivation for behavior change and has demonstrated positive effects on women drinkers. Objective: To assess the effectiveness of BI on increasing the motivation to change, drinking behavior in women attending PHC services with at-risk/harmful use of alcohol.

**Methods:** Randomized clinical trial with 20 women who screened positive for at-risk/harmful alcohol consumption using the Alcohol Use Disorder Identification Test (AUDIT) at a PHC service. Participants were randomly divided into two groups; the Intervention Group (IG) who participated in the BI (20 to 30-min individual session using motivational techniques) and the Control Group (CG) who received Brief Advice (BA) (feedback on the pattern of alcohol consumption). Outcomes were assessed at baseline and at follow‐ up after one and three months. For the intragroup and intergroup analysis, the generalized linear model was used, and for all tests, ≤ 0.05 value was considered significant.

**Results:** Data suggest the effectiveness of BI in the stages of readiness to change alcohol use (IG - pre-BI 4.89, post 1st BI 6.67, p = 0.12). The results observed on the CG (pre-BA 3.27, post 1st BA 3.09, p = 0.77), did not show significant pre/post increase in stages of readiness

**Conclusions:** BI delivered in a PHC setting has the potential to increase stages of readiness to change drinking behavior.

**Trial registration:** RBR65262c.

## A56 Systematic reviews of brief intervention in pregnancy: quality assessment with AMSTAR tool

### Vanderléa P. Cabral^1,2^, Ângela M. M. Abreu^3^, José M. B. Lima^4^, Carlos A. F. Andrade^5^

#### ^1^Fernandes Figueira National Institute/Fiocruz, Rio de Janeiro, RJ, Brazil; ^2^Anna Nery Rio de Janeiro Federal University, Rio de Janeiro, RJ, Brazil; ^3^Public Health Department, Anna Nery Rio de Janeiro Federal University, Rio de Janeiro, RJ, Brazil; ^4^Rio de Janeiro Federal University, Rio de Janeiro, RJ, Brazil; ^5^Department of Epidemiology and Quantitative Methods in Health, Oswaldo Cruz Foundation, Rio de Janeiro, RJ, Brazil

##### **Correspondence:** Vanderléa Poeys Cabral - poeys2008@gmail.com

*Addiction Science & Clinical Practice* 2019, **14(Suppl 1):**A56

**Background:** Drinking alcohol during pregnancy can cause harm to the woman and the fetus. Studies have shown promising results from brief interventions combined with the early detection of alcohol consumption in pregnancy in relation to the prevention of such damages. National and international guidelines have concluded that there is no safe dose of alcohol ingestion during pregnancy. The aim of this study is to assess the quality of the systematic reviews of brief interventions to avoid alcohol consumption among pregnant women.

**Methods:** We searched systematic reviews on brief interventions to avoid alcohol consumption by pregnant women via Medline, Cochrane Library and Cumulative Index to Nursing and Allied Health Literature (Cinahl). The quality of each systematic review was assessed using the validated tool Assessing the Methodological Quality of Systematic Reviews (AMSTAR).

**Results:** Six of 42 abstracts of systematic reviews identified were selected for full reading. Quality assessment was conducted in four studies. In two of these studies (Whitworth and Dowswell 2010; Stade et al. 2014), two reviewers independently completed the selection and data extraction. Both of them reached “high-quality” level (≥ 8/11 of the AMSTAR checklist). The first study authors’ (Whitworth and Dowswell) concluded that there is little evidence on the effects of pre-pregnancy health promotion. The second study authors’ (Stade et al.) concluded that the educational interventions may result in increased abstinence from alcohol, however, results were not consistent.

**Conclusions:** Although brief interventions are recommended to prevent prenatal alcohol use, there is currently insufficient evidence to recommend the widespread implementation of routine pre-pregnancy health promotion. These findings show that it is important to carry out systematic reviews and clinical trials of adequate quality.

## A57 Web-based screening and brief intervention with weekly text-message-initiated individualized prompts for reducing risky alcohol use among teenagers: focus groups and pilot study results

### Kristina Wille^1^, Silke Diestelkamp^1^, Rainer Thomasius^1^, ProHEAD Consortium^2^

#### ^1^German Center for Addiction Research in Childhood and Adolescence, University Medical Center Hamburg-Eppendorf, Hamburg, Germany; ^2^Heidelberg, Hamburg, Leipzig, Marburg, Schwäbisch Gmünd, Germany

##### **Correspondence:** Kristina Wille - k.wille@uke.de

*Addiction Science & Clinical Practice* 2019, **14(Suppl 1):**A57

**Background:** Technology based, short alcohol related interventions have the potential to reach a large number of recipients with a service that can be individualized. For young people in particular, technology based interventions represent an opportunity to address the target group in an age appropriate way and reach them in the context of their lives in the sense of an “ecological momentary intervention”. These are the results of two focus groups and pilot testing of viability, acceptance and functioning of the individualized SMS initiated boosters that had already been developed and are used in conjunction with web-based short interventions to reduce risky alcohol consumption in young people.

**Methods:** As part of the ProHEAD consortium, the effectiveness of fully automated web-based short interventions with weekly individualized SMS initiated boosters for schoolchildren under the age of 12 was investigated in a multi-center randomized controlled study (ProWISE-TIP study). Two focus groups (n = 5) of young people aged 13–17 were carried out to test the SMS initiated, fully automated booster messages. A group discussion based on guidelines was used to gather feedback on young people’s language, acceptance and on the content of the booster messages. Pilot testing (N = 20) of the functioning and viability of the SMS initiated individualized booster and the functioning of randomization for the 4-arm randomized controlled effectiveness study was carried out over 3 weeks with a final telephone survey.

**Results:** It was shown that the fully automated processes function technically and almost all participants used the SMS initiated individualized boosters over the three weeks. 50% of participants in the pilot study assessed the boosters as helpful with their alcohol use. 16.7% even stated that they drank less at the weekend due to the SMS messages. The tips for dealing with alcohol were found to be helpful and appropriate by almost 60% of participants.

## A58 Barriers and enablers for the implementation of the German Guideline on Screening, Diagnosis and Treatment of Alcohol-Related Disorders in routine care

### Christina Lindemann^1^, Maren Spiess^2^, Martin Härter^2^, Jens Reimer^1^, Uwe Verthein^1^, Bernd Schulte^1^, Angela Buchholz^2^

#### ^1^Centre for Interdisciplinary Addiction Research (ZIS), University Medical Centre Hamburg-Eppendorf, Hamburg, Germany; ^2^Department of Medical Psychology, University Medical Centre Hamburg-Eppendorf, Hamburg, Germany

##### **Correspondence:** Christina Lindemann - ch.lindemann@uke.de

*Addiction Science & Clinical Practice* 2019, **14(Suppl 1):**A58

**Background:** The German guideline on alcohol-related disorders aims to promote evidence-based care for the prevention and treatment of alcohol use disorders (AUDs) in different health care settings. As part of the Federal Ministry of Health’s funded project “Implementation and Evaluation of the Guideline on Screening, Diagnosis and Treatment of Alcohol-Related Disorders” (IMPELA), the objective of this research project was to identify barriers and facilitators for the guideline implementation in routine care.

**Method:** We followed a mixed-methods approach to analyze barriers and facilitators in different care settings in the city of Bremen. A questionnaire was distributed to relevant care providers as well as to patients with AUD. Based on the results of the survey, qualitative interviews were conducted to validate and deepen the quantitative findings.

**Results:** Data sets of 163 practitioners (63.2% female; 33.7% male) and 94 patients (42.3% female; 57.7% male) were analyzed. Main barriers expressed by the practitioners were a lack of knowledge about the guideline, limited time and financial resources for the provision of guideline-oriented AUD care, as well as long waiting periods before AUD treatment (47.8%). The most frequent barriers mentioned by patients were the fact that those affected did not seek help due to the fear of consequences or missing acceptance of their alcohol-related problems. Furthermore, patients stressed screening in areas other than health care (i.e. at the workplace) as well as direct referral between acute AUD care and rehabilitation as important areas of improvement. A potential facilitator to increase guideline-oriented care in the view of patients included public awareness campaigns. Practitioners reported to prefer setting specific summaries of the guidelines (e.g. fact-sheets, booklets).

**Conclusions:** The provider- and patient-related findings will be used to select and develop setting- and context-specific implementation strategies for specific recommendations of the German guideline on AUDs.

